# Inhibiting K63 Polyubiquitination Abolishes No-Go Type Stalled Translation Surveillance in *Saccharomyces cerevisiae*

**DOI:** 10.1371/journal.pgen.1005197

**Published:** 2015-04-24

**Authors:** Kazuki Saito, Wataru Horikawa, Koichi Ito

**Affiliations:** Department of Medical Genome Sciences, Graduate School of Frontier Sciences, The University of Tokyo, Kashiwa-city, Chiba, Japan; The University of North Carolina at Chapel Hill, United States of America

## Abstract

Incidental ribosome stalling during translation elongation is an aberrant phenomenon during protein synthesis and is subjected to quality control by surveillance systems, in which mRNA and a nascent protein are rapidly degraded. Their detailed molecular mechanisms as well as responsible factors for these processes are beginning to be understood. However, the initial processes for detecting stalled translation that result in degradation remain to be determined. Among the factors identified to date, two E3 ubiquitin ligases have been reported to function in distinct manners. Because ubiquitination is one of the most versatile of cellular signals, these distinct functions of E3 ligases suggested diverse ubiquitination pathways during surveillance for stalled translation. In this study, we report experimental evidences for a unique role of non-proteasomal K63 polyubiquitination during quality control for stalled translation. Inhibiting K63 polyubiquitination by expressing a K63R ubiquitin mutation in *Saccharomyces cerevisiae* cells markedly abolished the quality control responses for stalled translation. More detailed analyses indicated that the effects of K63R mutants were independent of the proteasome and that K63 polyubiquitination is dependent on Hel2, one of the E3 ligases. Moreover, a K63R ubiquitin mutant barely inhibited the quality control pathway for nonstop translation, indicating distinct mechanisms for these highly related quality control pathways. Our results suggest that non-proteasomal K63 polyubiquitination is included in the initial surveillance process of stalled translation and presumably triggers protein degradation steps upon translational stall. These findings provide crucial information regarding the detailed molecular mechanisms for the initial steps involved in quality control systems and their classification.

## Author Summary

Stalled translation during elongation is an aberrant phenomenon during protein synthesis. Thus, once detected, it is subjected to quality control in which mRNA and a nascent protein are rapidly degraded. Although the mechanism of degradation for stalled translation is reasonably well understood, the initial processes, including those for detecting stalled translation, have not been determined. The ubiquitin proteasome pathway has been determined to function in the degradation of a nascent protein during stalled translation. Because a ubiquitin signal is one of the most versatile of cellular signals, we investigated the roles of various ubiquitination mechanisms in the budding yeast *Saccharomyces cerevisiae* using ubiquitin mutants that inhibited the polymerization of specific ubiquitin chains. We identified a role of non-proteasomal K63 polyubiquitination in stalled translation surveillance. Moreover, a K63R ubiquitin mutant barely inhibited the quality control pathway for nonstop translation, indicating distinct mechanisms for these highly related quality control pathways. These findings provide insights into the fundamental mechanisms for the initial processes of stalled translation surveillance and further emphasize the versatility of ubiquitin signals in cellular systems.

## Introduction

In addition to accurate protein synthesis in the ribosome, translational quality control pathways make significant contributions for appropriate gene expression [1]. Once aberrant mRNA templates, such as those with premature stop codons or those without stop codons, are detected during protein synthesis, mRNAs and nascent proteins are rapidly degraded by nucleases and the proteasome as quality controls pathways [2]. Stalled translation during elongation is also recognized as an aberrant translation that is subject to quality control [3]. A robust secondary RNA structure [4], consecutive polybasic amino acids [5], and rare codons [6] have been reported to induce strong translational stalling that is potentially susceptible to quality control surveillance.

The quality control for stalled translation surveillance involves a complex machinery, which includes a number of complexes and factors. mRNA degradation is initiated by an endonuclease, which remains to be identified, and proceeds via the actions of exonucleases. Kem1/Xrn1, a component of a processing body for mRNA turnover, is responsible for 5’ to 3’ mRNA degradation and a multiprotein complex, a so-called exosome, functions in 3’ to 5’ mRNA degradation in association with a Ski complex and Ski7 [3]. A complex of Dom34/Hbs1, which forms a structure that mimics tRNA/EF1α [7], functions in the disassembly of a stalled ribosome [3, 8]. A scaffold protein, Asc1, was also shown to be involved in the stalled translation surveillance by a gene knockout analysis, although its precise role is unknown [9, 10]. In addition, E3 ubiquitin ligases and their associated factors such as Rqc1 have been identified [10, 11]. Although numerous factors have been identified, the initial steps in stalled translation surveillance, such as detection of aberrance and triggering of subsequent degradation, have not been revealed.

Two E3 ligases, Hel2 and Ltn1, function in the quality control for stalled translation in apparently distinct manners [12]. Hel2 was reported to function in histone ubiquitination [13] and deleting the Hel2 gene resulted in enhanced expression of a full-length protein from mRNA with a stall signal in the midst of its reading frame [10]. Ltn1 was reported to be involved in polyubiquitination for proteasomal degradation [11]. A global analysis of cotranslational ubiquitination suggested that Hel2 and Ltn1 function in distinct manners [14]. However, the distinct roles of these E3 ligases remain unclear.

Ubiquitination is one of the most versatile cellular signals because polyubiquitin can be synthesized by linkage at a specific lysine or N-terminal methionine residues in various cellular processes [15]. In addition to polyubiquitination, monoubiquitination has been reported to function as a cellular signal [16, 17], which further establishes the versatility of ubiquitin signals. Some regulatory pathways are governed by multiple ubiquitin signals. For example, NF-κB activation is regulated by at least K11, K48, K63, and linear polyubiquitin chains [18].

In this report, we demonstrate a unique role of non-proteasomal K63 polyubiquitin in the quality control for stalled translation in the budding yeast *Saccharomyces cerevisiae* using a number of ubiquitin mutants that interfere with specific polyubiquitination at each lysine residue. Inhibiting K63 polyubiquitination resulted in the expression of a full-length product from mRNA with a stall signal and the K63 polyubiquitination is dependent on Hel2, which provided insights into the initial processes involved in stalled translation surveillance. This same ubiquitin mutant barely affected the quality control for nonstop translation, suggesting that initial steps are distinct between these two related quality control pathways.

## Results

### Distinct roles of Hel2 and Ltn1 E3 ubiquitin ligases in the quality control for stalled translation

**Fig 1:**
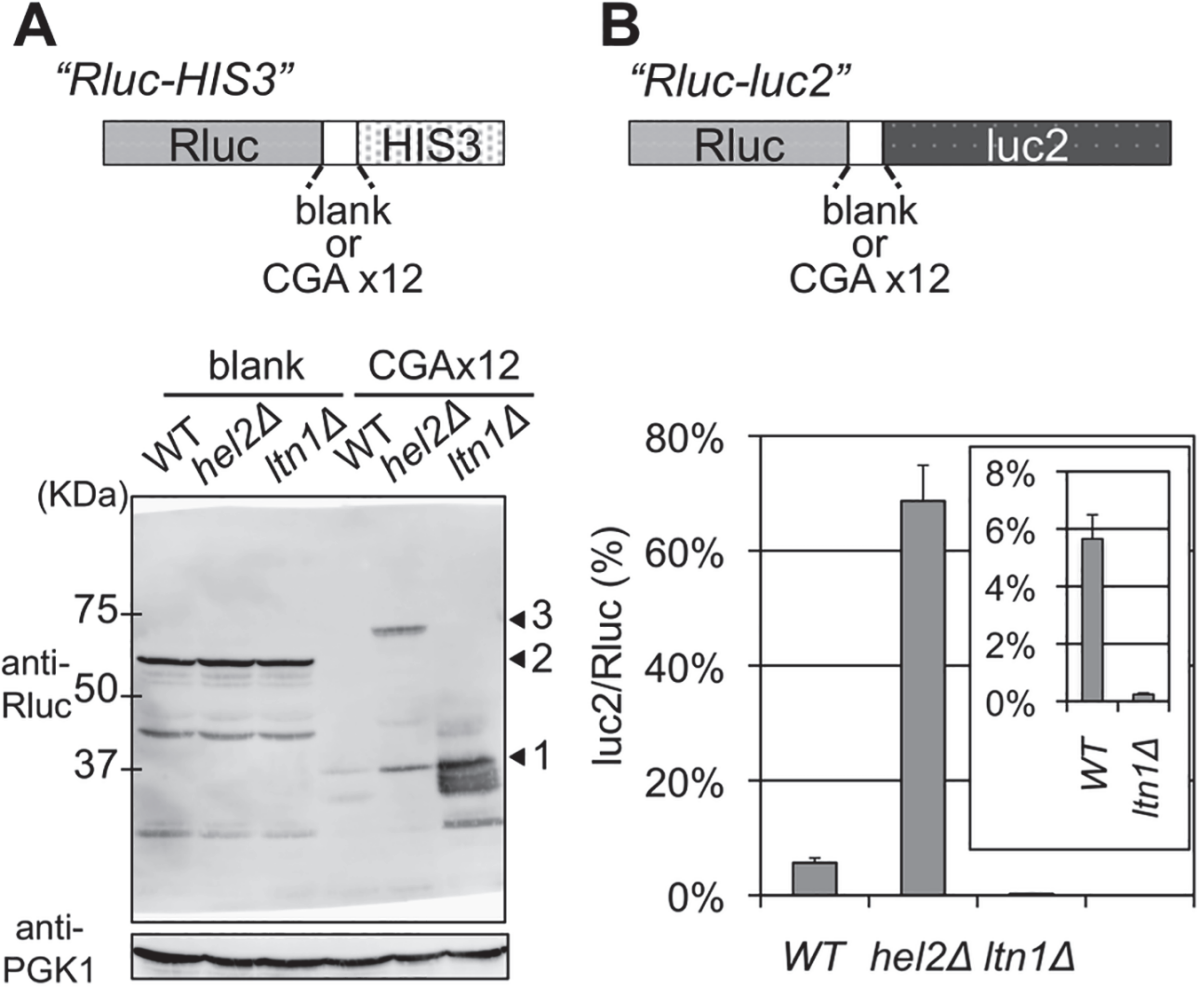
Comparisons of the effects of deleting Hel2 and Ltn1. (A) Western blot analysis for Rluc-HIS3 reporters from the wild-type (WT), *hel2* ***∆***, and *ltn1* ***∆*** strains (BY4727, SKY61, S18-E01). A schematic image of reporter fusion genes, Rluc-HIS3, either with or without 12 CGA codon repeats, is illustrated above. The expression of fusion protein was detected using a Rluc antibody. PGK1 was used as a loading control. Arrowhead 1 indicates the Rluc protein alone, arrowhead 2 indicates the Rluc-blank-HIS3 protein, and arrowhead 3 indicates the Rluc-CGA x12-HIS3 protein. (B) Dual luciferase assay for Rluc-CGA x12-luc2 reporter in the wild-type (WT), *hel2* ***∆***, and *ltn1* ***∆*** strains (HRKW-2, SKY113, HRKW-6). A schematic image of reporter fusion genes, Rluc-luc2, either with or without 12 CGA codon repeats, is illustrated above. Bars indicate luc2/Rluc ratios. Percentages were standardized using the results of a dual luciferase assay for Rluc-blank-luc2 in the wild-type strain (HRKW-1). The inset indicates the results with the *ltn1* ***∆*** strain with an appropriately adjusted range. Average luc2/Rluc ratios and standard deviations were determined from three independent measurements.

A CGA (arginine) codon was reported to be an exceptional rare codon that caused a profound translational stall in the budding yeast *S*. *cerevisiae* [19]. By utilizing this property, we conducted genetic screenings to identify those factors that could function during the early stages of quality control for stalled translation at consecutive CGA (arginine) codons. For this purpose, we constructed a reporter system using 12 CGA codons inserted ahead of a reporter gene (hereafter, CGA reporter) and a reference reporter without this CGA repeat (blank reporter) (Fig 1A). Any functional defects in the quality control for stalled translation due to the genetic alterations of the responsible genes could be detected by facilitated growth due to the enhanced expression of the reporter gene.

In our genetic screening with this consecutive CGA reporter system, we identified an ubiquitin E3 ligase, Hel2, as a factor for the quality control of our CGA stalled translation system (S1 Fig). In a previous report, Hel2 was also reported to be involved in the quality control for stalled translation caused by a consecutive lysine [10]. Thus, this result suggested a common role of Hel2 in two different types of the quality control for stalled translation.

However, the genetic analysis with our CGA reporter system did not detect any effects by Ltn1 (S1 Fig), which is also an ubiquitin E3 ligase that has been reported to function in stalled translation [10]. These results clearly suggested distinct roles of the two E3 ubiquitin ligases, Hel2 and Ltn1, in the quality control system for stalled translation, as previously reported [10, 12].

Western blot analysis for the reporter construct in the *ltn1* ***∆*** strain detected a highly elevated expression of stalled Rluc protein, which is situated upstream of the CGA stall signal (Fig 1A), due to the inhibition of rapid proteolysis of stalled products, as previously reported [10, 11]. In contrast, comparable amounts of full-length proteins and the stalled Rluc protein were detected in the *hel2* ***∆*** strain (Fig 1A) consistently as previously reported [12].

Next, for quantitative analysis, an alternative assay construct was introduced, in which the reporter HIS3 was replaced with firefly luciferase (luc2), as previously reported [12, 20]. The expressions of both the full-length and stalled proteins could be monitored by a dual luciferase method (Fig 1B). These results were recorded as percentages of luc2/Rluc ratios. The luc2/Rluc ratios for the conditions of interest were standardized by the luc2/Rluc ratio derived from the blank reporter in a wild-type strain, which was set at 100% (S2A Fig). The results for the blank reporter were equivalent to 100% for the wild-type, *ltn1* ***∆*** and *hel2* ***∆*** strains (S2A Fig). For the wild-type strain, the average luc2/Rluc ratio due to the CGA reporter was 5.66% (Fig 1B and S1 Table). The average with the *ltn1* ***∆*** strain was 0.24%, which was much lower than that with the wild-type strain and consistent with the specific enrichment of the stalled Rluc product detected by western blotting (Fig 1A). In comparison, the average with the *hel2* ***∆*** strain was increased to 68.66% (Fig 1B). This indicated a marked increase in the full-length product Rluc-luc2 compared with the stalled product Rluc. This again provided evidence for distinguishable mechanisms in which these two E3 ligases participated, consistently as previously reported [10, 12]. These results prompted us to investigate the temporal and mechanistic roles of ubiquitination in the quality control for stalled translation.

### Non-proteasomal K63 polyubiquitination involvement in the quality control for stalled translation

**Fig 2:**
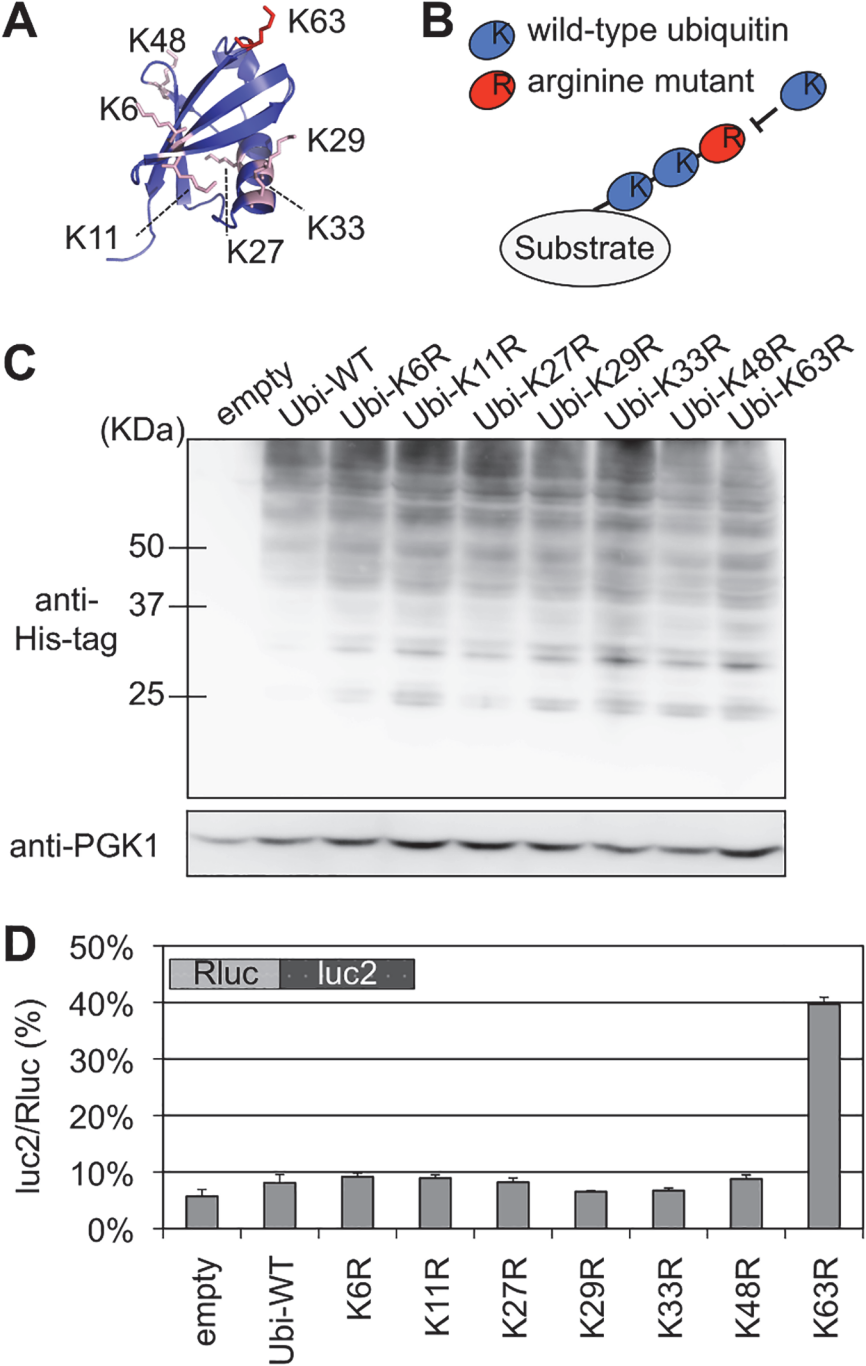
Effect of K63 polyubiquitination on the quality control for stalled translation. (A) Structure of Ubiquitin protein and location of the lysine residues for polyubiquitination [id: 1UBQ]. K63 (red) and other lysine residues (pink) are shown in sticks. The structural model was generated using MolFeat version 3.5 (Fiatlux, Tokyo, Japan). (B) Schematic illustration for the inhibition of polyubiquitination by arginine mutants. Arginine mutants were expressed from expression plasmids in yeast cells harboring endogenous ubiquitin genes on its genome. An incorporation of an arginine mutant into the (poly-) ubiquitin chain terminates further elongation. (C) Cellular ubiquitination levels of wild-type and ubiquitin mutants with a single arginine substitution (K6R, K11R, K27R, K29R, K33R, K48R, K63R). Ubiquitin genes were fused with His-tag and expressed from plasmids in the wild-type strain (BY4727). Cellular ubiquitination levels were detected by antibody for His-tag. PGK antibody was used as loading control. (D) Dual luciferase assay for the effects of arginine mutants of ubiquitin protein on the Rluc-CGA x12-luc2 reporter. Plasmids that included ubiquitin arginine mutants were introduced into a wild-type strain (HRKW-2). Ubi-WT indicates wild-type ubiquitin. Average luc2/Rluc ratios and standard deviations were determined from three independent measurements.

We constructed a series of ubiquitin mutants to investigate the involvement of specific type of polyubiquitination linkages in the Hel2 related quality control of stalled translation, particularly in the reduction of a full-length product. Each of these ubiquitin mutants had an arginine substitution at one of the lysine residues for a specific polyubiquitination linkage (Fig 2A). Thus, incorporation of these mutants in a polyubiquitin chain inhibits further elongation and results in substantial reduction of polyubiquitination effect (Fig 2B) [21–23]. These ubiquitin mutants were overexpressed in yeast strains with stalled translation reporters. The cellular expression levels of ubiquitin were shown to be relatively comparable to each other (Fig 2C).

**Fig 3:**
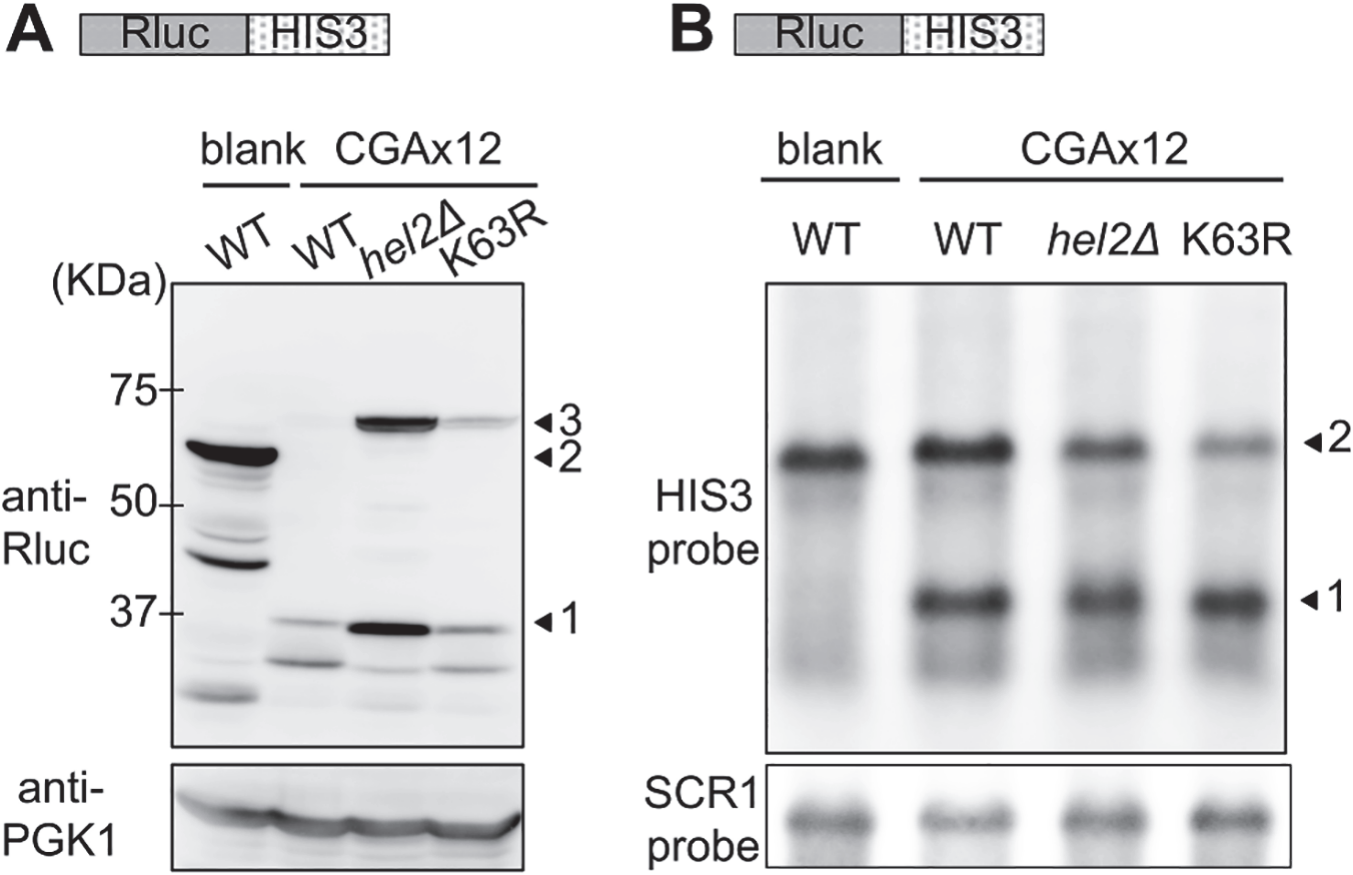
Comparison of the effects of Hel2 deletion and K63R expression on protein and mRNA levels in stalled translation. (A) Western blot analysis for the effects of K63R ubiquitin on Rluc-HIS3 reporter. blank indicates the result of the Rluc-blank-HIS3 reporter, and CGAx12 indicates the results of the Rluc-CAAx12-HIS3 reporter. WT indicates the wild-type strain (BY4727) without K63R ubiquitin, *hel2∆* indicates the *hel2∆* strain (SKY61), and K63R indicates the wid-type strain expressing K63R ubiquitin. Arrowhead 1 indicates the Rluc protein alone, arrowhead 2 indicates the Rluc-blank-HIS3 protein, and arrowhead 3 indicates the Rluc-CGA x12-HIS3 protein. PGK antibody was used as loading control. (B) Northern blot analysis for the effects of K63R ubiquitin on Rluc-HIS3 reporter. blank and CGAx12 as well as WT, *hel2∆*, and K63R are as in (A). The reporter mRNA was probed by HIS3 complementary DNA fragment. Arrowhead 1 indicates the cleaved HIS3 mRNA and arrowhead 2 indicates the full-length mRNA. SCR1 mRNA was detected as a loading control.

Although these ubiquitin mutants barely affected the luc2/Rluc ratios of blank reporter (S2B Fig), expression of the K63R ubiquitin mutant markedly increased the luc2/Rluc ratio of the CGA reporter construct to 39.7% (Fig 2D and S1 Table). Consistently expression of K63R ubiquitin enhanced the colony growth on the assay plates (S3 Fig). Furthermore as shown by western blot analysis (Fig 3A), the expression of full-length reporter protein, which appeared at the same position of the full-length product in the *hel2∆* strain, was increased by the expression of K63R ubiquitin (Fig 3A lane K63R). Contrastingly, expression of the full-length mRNA was less markedly affected by deletion of Hel2 and expression of K63R as compared with expression of the full-length protein (Fig 3B), suggesting that K63 polyubiquitination as well as Hel2 have more direct roles on protein turnover.

**Fig 4:**
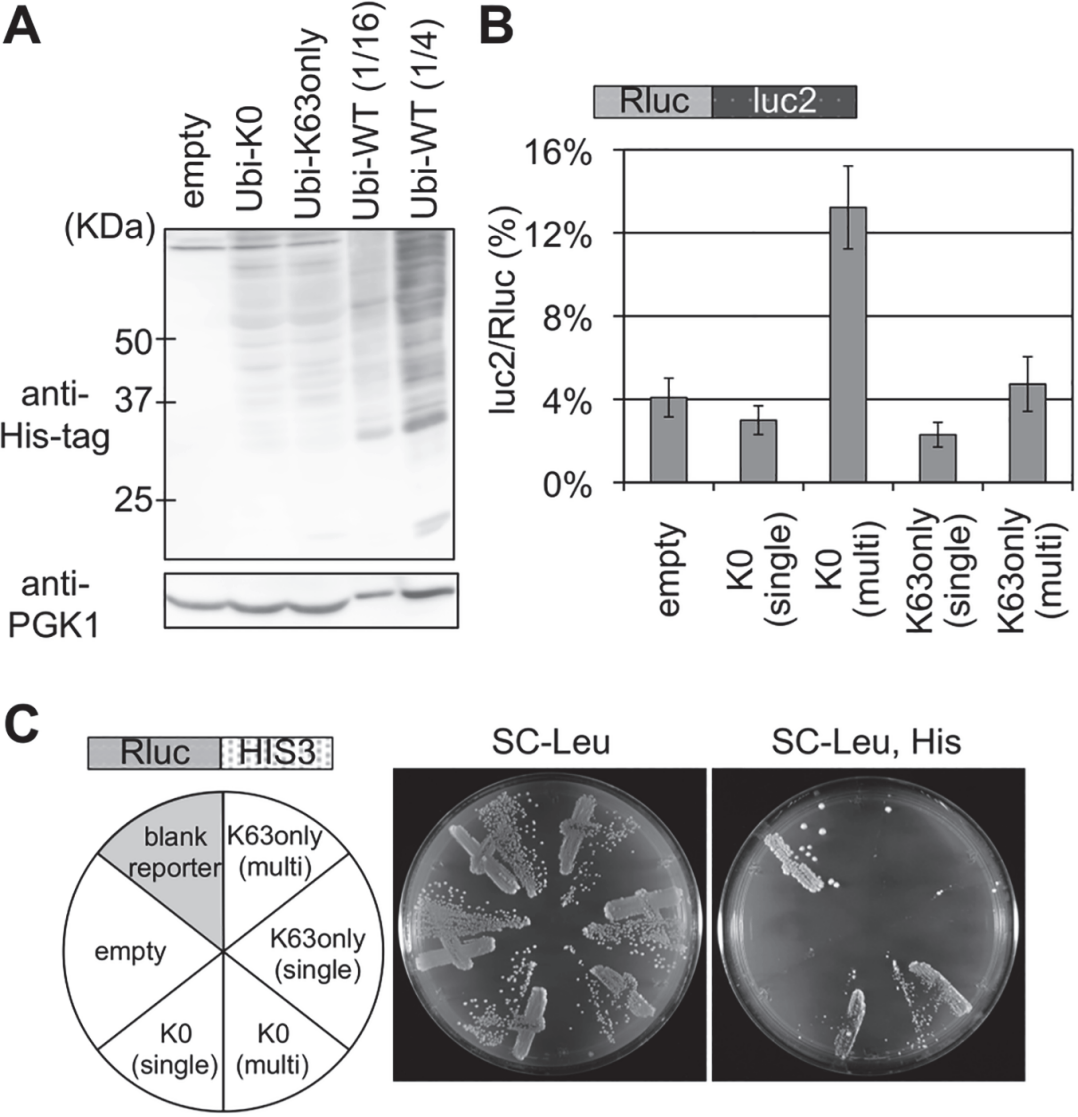
Effects of K0 and K63only ubiquitin mutants on stalled translation. (A) Cellular ubiquitination levels of K0 and K63only ubiquitin mutants. Ubiquitinated proteins were detected as above. Amount of the protein sample from a cell expressing His-tagged wild-type ubiquitin are decreased to 1/16 and 1/4 dilution. (B) Dual luciferase assay for the of K0 and K63only ubiquitin mutants on the Rluc-luc2 reporter. Plasmids bearing K0 or K63only ubiquitin mutants were introduced into a wild-type strain harboring Rluc-luc2 reporter strain (HRKW-2). (single) indicates the transformant with ubiquitin expression from single copy plasmids, and (multi) indicates that from multiple copy plasmids. Average luc2/Rluc ratios and standard deviations were determined from three independent measurements. (C) Genetic colony growth test for the effect of K0 and K63only ubiquitin mutants on the Rluc-HIS3 reporter (SKY24). Types of ubiquitin vectors are indicated on the left. Transformant colonies were streaked on the SC-Leucine (Leu) (middle), and SC-Leucine, Histidine (Leu, His) (right) plates, and colony growth was monitored for 4 days at 30°C.

To further confirm the role of K63 polyubiquitin, we tested two additional ubiquitin mutants, one is K0 ubiquitin, in which all of the lysine residues are substituted to arginine, and the other is K63only ubiquitin, in which all of the lysine residues except K63 are substituted to arginine, thus K63 serves as a solo lysine for polyubiquitination. The cellular ubiquitination levels of K0 ubiquitin and K63only ubiquitin were substantially lower than wild-type ubiquitin (Fig 4A), therefore both ubiquitin mutants were also expressed from multicopy plasmid. Although the effect was less marked than K63R ubiquitin presumably due to the cellular ubiquitination level, K0 ubiquitin expressed from the multicopy plasmid increased the luc2/Rluc ratio (Fig 4B and S1 Table). In the colony growth assay, K0 ubiquitin on both plasmids induced significant growth on a plate lacking histidine (Fig 4C). Contrastingly, K63only ubiquitin on neither of the plasmids affected at all (Fig 4B and 4C), suggesting that the K63 solely is sufficient to abolish the inhibitory effect of K0 ubiquitin. These results suggested that the K63 polyubiquitin signal is predominantly responsible for the reduction of a full-length product in the quality control.

In order to test the roles by K63 polyubiquitination in stalled translation on other stall signals, several additional stall signals such as poly CGA, poly lysine, poly arginine and poly GGN, as in preceding reports [5, 24], are inserted between the luciferase genes. As shown in S4 Fig (luc2/Rluc ratios of WT columns), stall effects of most of the additional signals were much weaker than that of the CGA signal, so as the effect of the genetic variants (S4 Fig and S2 Table), resulting in low significance scores. Nevertheless, importantly, the elongated consecutive polylysine signal, polylysine (x24), significantly increased the stall effect. For this polylysine (x24), luc2/Rluc ratios were markedly decreased by deletion of Ltn1 and the ratio increased by deletion of Hel2 as well as K63R expression, suggesting the involvement of Hel2 and K63 polyubiquitination for the quality control of this stall signal, consistently with the previous reports [10, 11].

**Fig 5:**
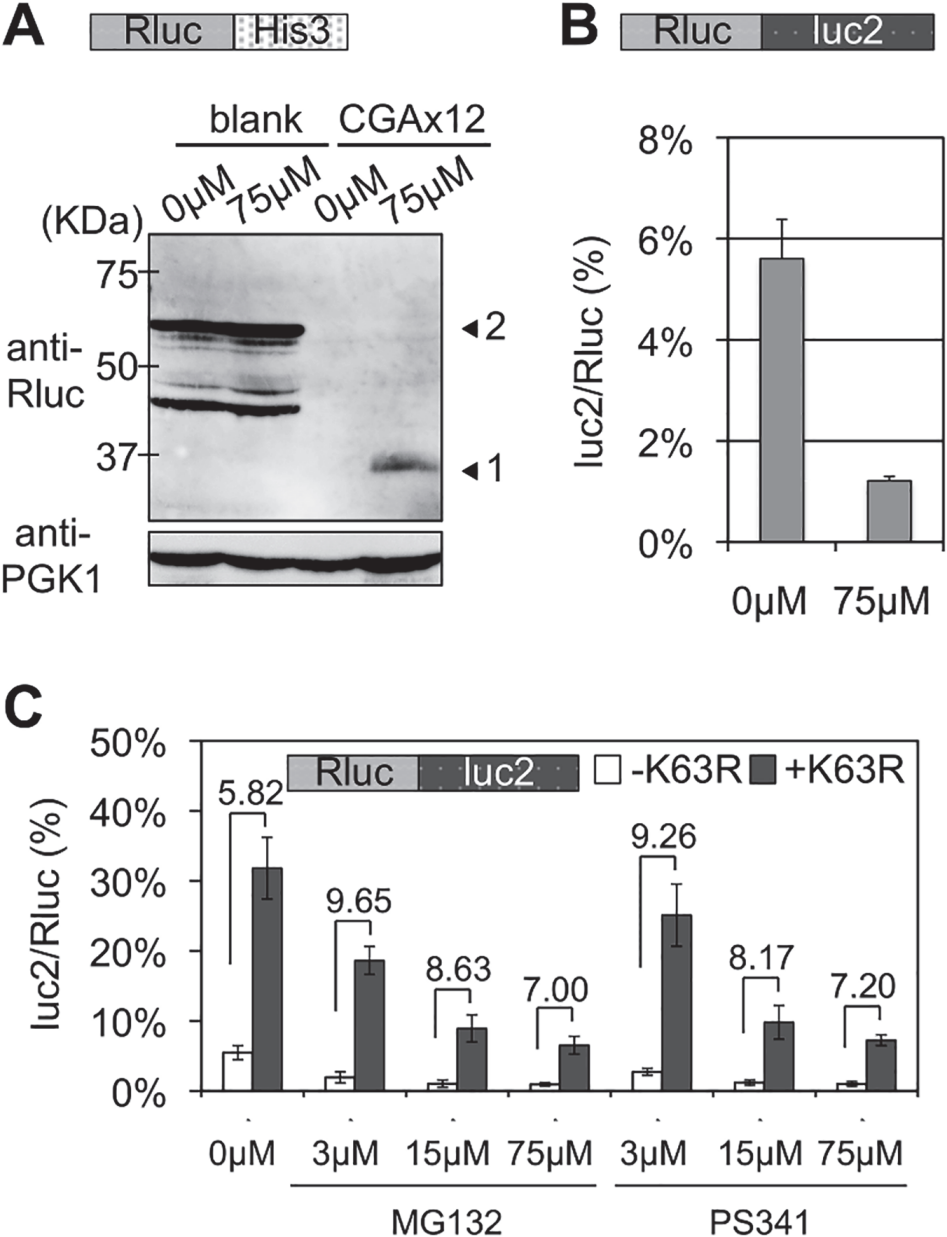
Effects of proteasome inhibitors, MG132 and PS341, on stalled translation and K63R expression. (A) Western blot analysis for effect of MG132. Vectors with Rluc-HIS3 reporters were introduced into a wild-type strain (BY4727). Yeast transformants were incubated for 5 h with either DMSO (e.g., 0 μM MG132) or 75 μM MG132. Arrowhead 1 indicates the Rluc protein only and arrowhead 2 indicates the Rluc-blank-HIS3 protein. PGK1 was used as a loading control. (B) Dual luciferase assay for the effect of MG132 on wild-type strain with Rluc-CGA x12-luc2 reporter (HRKW-2). MG132 was used as in (A). (C) The empty or K63R ubiquitin mutation plasmids were introduced into the *pdr5* ***∆*** yeast strain harboring Rluc-luc2 reporter (SKY142). The transformants were incubated in the presence of MG132 or PS341 at indicated concentrations for 2 h. Fold differences between the percentages of luc2/Rluc ratios between the transformants with the absence (-K63R) or presence (+K63R) of K63R expression are indicated over the top of the bar graphs.

One of the primary functions of polyubiquitination is to induce proteolysis by the proteasome [15, 25]. Thus, we tested the effects of the proteasome inhibitor, MG132. Addition of MG132 gave similar results to those by deleting Ltn1, as previously reported [11], i.e., enhanced expression of upstream Rluc protein (Fig 5A) and a decrease in the luc2/Rluc ratio (Fig 5B and S1 Table). These effects of MG132 indicated that the proteasome was irrelevant for the expression of a full-length protein from stalled translation. Thus, this suggests that the function of a K63 polyubiquitin chain for the quality control of a stalled ribosome is not mediated by the proteasome. Consistently, addition of MG132 and another proteasome inhibitor, PS341, at different concentrations did not markedly alter the fold differences in luc2/Rluc ratios between transformants of an empty vector (-K63R) and a K63R ubiquitin mutant (+K63R) (Fig 5C and S1 Table). Yeast strains that expressed only K63R ubiquitin were reported to be sensitive to anisomycin [26]. Consistent with this, overexpressing our K63R ubiquitin mutant increased the sensitivity to anisomycin (S5A Fig), indicating that our K63R ubiquitin mutant was valid. Taken together, these results indicated that K63 polyubiquitination functions as a non-proteasomal signal to inhibit the expression of a full-length protein from stalled translation.

### Temporal order of K63 polyubiquitin signals during the quality control for stalled translation

**Fig 6:**
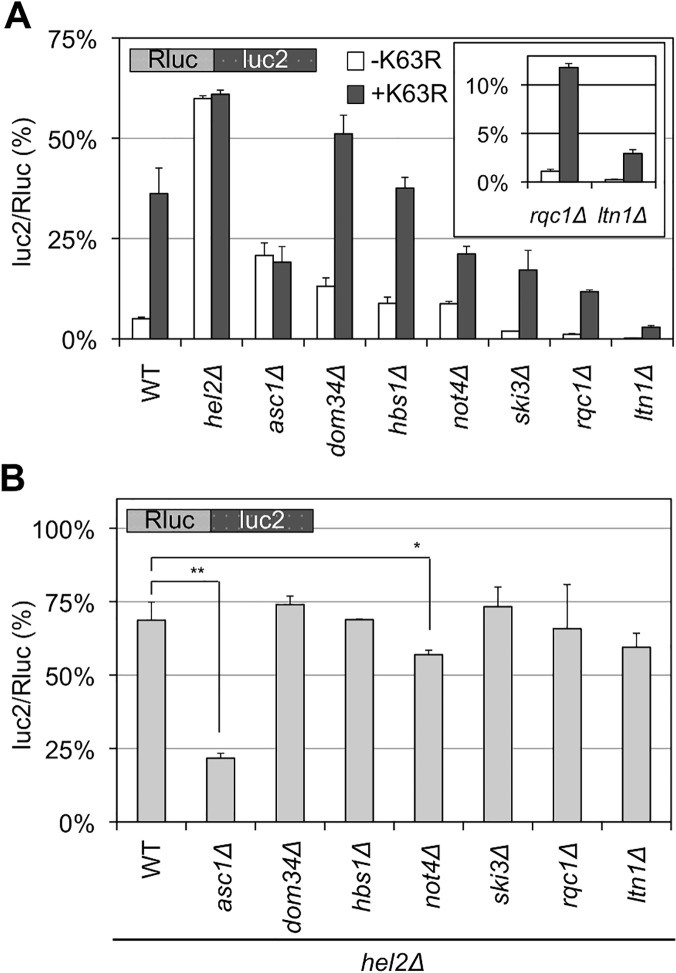
Temporal regulation of K63 polyubiquitination and Hel2 with other factors for stalled translation. (A) The luc2/Rluc ratios for Rluc-luc2 reporter in the wild-type (WT) and single knockout strains (as indicated) in the presence (+K63R) or absence (-K63R) of K63R expression. The inset shows the results for the *rqc1* ***∆*** and *ltn1* ***∆*** strains. The average luc2/Rluc ratios and their standard deviations were obtained for three independent measurements. (B) The luc2/Rluc ratios for Rluc-luc2 reporter in the strains with double knockout of Hel2 and other factors involved in stalled translation. All strains are *hel2* ***∆***. Thus, as examples, WT in this figure indicates a *hel2* ***∆*** single knockout strain, and *asc1* ***∆*** indicates the *hel2* ***∆*** *asc1* ***∆*** strain. The average luc2/Rluc ratios and their standard deviations were obtained for three independent measurements. *p < 0.05, **p < 0.01.

The quality control for stalled translation involves factors other than Hel2 and Ltn1, such as Not4, Asc1, Dom34, Hbs1, a Ski complex, and a RQC complex. Deleting these factors did not markedly affect the ratio when using a blank reporter (S2C Fig). Then, as shown in Fig 6A, deleting these factors either decreased or increased the luc2/Rluc ratio with our CGA reporter (“-K63R” in Fig 6A and S1 Table), as previously reported [3, 9, 10]. Among them, the results of *not4∆* strains seem somewhat inconsistent among previous reports [5, 11, 14, 27], presumably reflecting multiple roles of Not4 in gene expression and strain variability as previously discussed [14, 27]. The *not4∆* strains in this study exhibited slower growth on YPD medium, as well as high sensitivity to azetidine-2-carboxylic acid (AZC) (S6 Fig), as previously reported [14, 27], supporting the validities of our strains.

Next, the luc2/Rluc ratios in those genetic backgrounds were monitored in the presence of K63R ubiquitin to analyze the order of action of K63 polyubiquitination. In the *asc1* ***∆*** and *hel2* ***∆*** strains, K63R expression barely affected the luc2/Rluc ratios (Fig 6A), whereas in other strains, the luc2/Rluc ratios were increased to the expression of K63R (Fig 6A). The temporal relationships of Hel2 with respect to other factors were also analyzed by determining the luc2/Rluc ratios of double knockout strains with Hel2 (Fig 6B and S1 Table). The *hel2* ***∆*** *asc1* ***∆*** strain exhibited a luc2/Rluc ratio of 21.75% (Fig 6B), which was close to the ratio with the *asc1* ***∆*** single knockout strain (19.23%; S7 Fig), whereas other strains had rather comparable ratios (60%–74%) close to the *hel2* ***∆*** single knockout strain (68.66%; Fig 6B) except for the *not4∆hel2∆* strain (discussed later). For comparisons, the luc2/Rluc ratios of double knockout strains with Asc1 were examined, except for *not4∆asc1∆* strain, which could not be constructed in our strain background. All strains with *asc1∆* background exhibited comparable luc2/Rluc ratios (S7 Fig and S1 Table). Further, the *hel2∆* and *asc1∆* strains exhibited hyper-sensitive growth to anisomycin (S5B Fig). Anisomycin inhibits translation elongation [28], thus anisomycin sensitivities induced by expression of K63R ubiquitin and deletion of Hel2 and Asc1 supported the functional relevance of Hel2, Asc1, and K63 polyubiquitination in the quality control of stalled translation.

These results suggest that the effect of K63 polyubiquitination in this study depends on the functions of Hel2 and Asc1. And the K63 polyubiquitination act independently with other factors, such as Dom34/Hbs1 complex, Ski3, Ltn1, and Rqc1. It was also indicated that Asc1 functions prior to these other factors, including Hel2, for stalled translation surveillance (Fig 6B and S7 Fig). The early functions of Asc1 and Hel2 were consistently suggested by the expression of a full-length protein from a polylysine reporter in previous report [10].

### Differential roles of putative K63 polyubiquitin signals in two related quality control pathways

Next, we investigated the effects of K63 polyubiquitination and Hel2 on the quality control for nonstop translation. mRNA without an in-frame stop codon is rapidly degraded by exosomes and a Ski complex in an mRNA surveillance system, called nonstop mRNA decay (NSD) [29]. A nascent protein derived from nonstop mRNA is rapidly degraded by the ubiquitin proteasome pathway in collaboration with the E3 ligase Ltn1 [11]. A Dom34/Hbs1 complex was also reported to function during nonstop translation [24]. Because many factors participate with similar roles in the quality control pathways for both nonstop and stalled translations, the distinction between these pathways is ambiguous.

**Fig 7:**
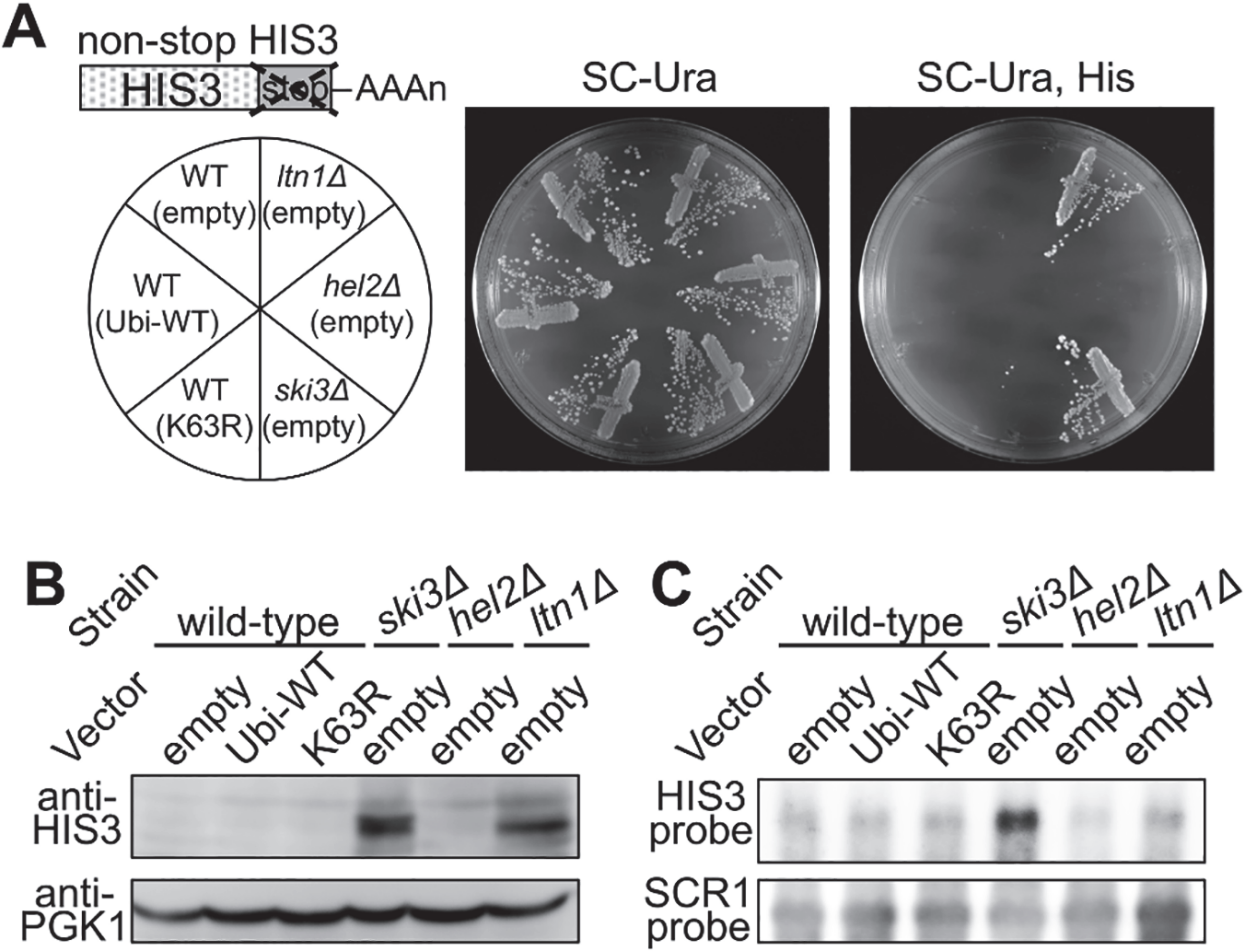
Effects of K63 polyubiquitination and Hel2 on quality control for non-stop translation. (A) Genetic colony growth test for the nonstop HIS3 reporter. A schematic image of a nonstop HIS3 allele is illustrated above. Empty vector (empty), wild-type ubiquitin (Ubi-WT), or K63R ubiquitin (K63R) plasmids were introduced into the wild-type, *ski3* ***∆***, *hel2* ***∆***, or *ltn1* ***∆*** strains harboring nonstop HIS3 reporter (S17-A08, S17-A09, SKY123, SKY137). Types of knockout alleles and vectors are indicated on the left. Transformant colonies were streaked on the SC-Uracil (Ura) (middle), and SC-Uracil, Histidine (Ura, His) (right) plates, and colony growth was monitored for 3 days at 30°C. (B) Western blot analysis for the nonstop HIS3 reporter. Protein samples were extracted from the transformants of the nonstop HIS3 reporter strains as in (A). The expression of HIS3 protein was detected using a HIS3 polyclonal antibody. PGK1 was used as a loading control. (C) Northern blot analysis for the nonstop HIS3 reporter. mRNA samples were extracted from the transformants of the nonstop HIS3 reporter strains as in (A). The nonstop HIS3 reporter mRNA was probed by HIS3 complementary DNA fragment. SCR1 mRNA was detected as a loading control.

A nonstop HIS3 reporter allele was employed for the evaluation of the role of K63 polyubiquitin in nonstop translation (Fig 7A) [29]. Nonstop HIS3 mRNA and its nascent product are substrates for nonstop translation surveillance, and thus, expression of HIS3 enzyme from the nonstop mRNA is not sufficient for cell viability in a medium without histidine. Upon disrupting the genes responsible for nonstop translation surveillance, the strains restore sufficient expression of HIS3 enzyme for normal growth.

The nonstop HIS3 allele was integrated into the genomes of a *his3* ***∆*** strain as well as Ski3, Hel2, and Ltn1 deletion strains, and then their cellular expressions were assessed by histidine auxotrophic growth, western blotting, and northern blotting. The *ski3* ***∆*** and *ltn1* ***∆*** strains were viable on a plate that lacked histidine, whereas the K63R ubiquitin transformant and *hel2* ***∆*** strain were not viable (Fig 7A). Western blot showed increased HIS3 protein expression in the *ski3* ***∆*** and *ltn1* ***∆*** strains but not in the K63R ubiquitin transformant or the *hel2* ***∆*** strain (Fig 7B). Also, northern blot analysis showed that nonstop HIS3 mRNA was stabilized in the *ski3∆* strain and the mRNA level did not increased in the *ltn1∆* strain, as reported previously [30]. The deletion of Hel2 and expression of K63R ubiquitin hardly affected the stability of the reporter mRNA (Fig 7C). These results indicated that K63 polyubiquitination and Hel2 are not involved in the quality control for nonstop translation.

To confirm this speculation further, we tested the involvement of K63 polyubiquitination and Hel2 in another type of nonstop translation derived from self-cleavage of hammerhead ribozyme inserted in reporter gene [31, 32]. Except for Ski7 [31], it is reported that quality control of ribozyme-derived nonstop translation is accomplished by common factors with the nonstop mRNAs with polyA tail [24, 31, 33]. Hammerhead ribozyme sequence was inserted at the junction of a fusion gene of Rluc and HIS3 gene (S8A Fig) and expression of Rluc protein was monitored by western blot. Similarly to the results of nonstop allele of HIS3, upstream Rluc protein derived from self-cleaved mRNA was detected in the *ski3∆* and *ltn1∆* strains, but overexpression of K63R and deletion of Hel2 hardly enhanced the level of Rluc protein (S8B Fig).

Recently, Hel2 had been reported to weakly affect the quality control of nonstop translation [14] and this is inconsistent to our speculation mentioned above. Taking the high complexity of the quality control systems as well as the yet-unknown cryptic factors behind the genetic backgrounds into consideration, it is possible to speculate that the inconsistency arises from certain masking effects by other factors collaborating with K63 polyubiquitination and Hel2 in the quality control of nonstop mRNA.

Collectively with two type of reporter genes, the nonstop HIS3 allele and the hammerhead derived nonstop mRNA, our results suggested that K63 polyubiquitination as well as Hel2 have much more significant roles on the quality control of stalled translation that occurs during elongation.

## Discussion

During the quality control for stalled translation, at least two E3 ubiquitin ligases, Ltn1 and Hel2, are distinctly involved (Fig 1) as reported previously [10, 12]. Ubiquitin is used for versatile cellular signals through polymerization via its lysine residues [15]. In this study, using ubiquitin mutants with Lys-to-Arg substitutions, which inhibit a specific type of polymerization linkage, we identified a unique role of K63 polyubiquitination in stalled translation surveillance in budding yeast *S*. *cerevisiae* (Figs 2, 3, 4 and S3). In stalled translation, the K63 polyubiquitination did not serve as a proteasomal signal (Fig 5), functioned in the presence of Hel2 and Asc1, and was independent of other known steps (Fig 6A). These findings suggest that multiple types of polyubiquitination signals and ubiquitin ligases are involved in the quality control for stalled translation, i.e., K48 polyubiquitination for proteasomal degradation and K63 polyubiquitination in this study. Our findings of K63 polyubiquitination provide important clues to elucidate the mechanisms of stalled translation surveillance and for novel roles of ubiquitin signals in cellular systems.

K63 polyubiquitination involvement in various pathways, such as those for cell signaling, permease trafficking, and DNA repair, have been extensively investigated [34–36]. In these pathways, a K63 polyubiquitin chain recruits factors for succeeding reactions through direct recognitions by K63 polyubiquitin specific receptors [37–41]. In this study, because K63 polyubiquitination was not involved in proteasomal degradation (Fig 5), it can be assumed that K63 polyubiquitination in stalled translation functions in recruiting related factors, and thereby serves as an intermediate between the recognition of stalled translation and degradation and/or dissociation of stalled machineries. In this scenario, a receptor of the K63 polyubiquitin chain for stalled translation must play an important role in transmitting a ubiquitin signal to subsequent reactions.

It has been reported that the rate of translation elongation is extensively regulated for a variety of reasons [42–44]. Therefore, a quality control surveillance system must be able to distinguish between aberrantly stalled states that are to be eliminated and rather slow translations that should be normally maintained. It was also reported that the length of a polyubiquitin chain was crucial for efficient recognition by its receptors [40–41]. In many cases, the length of a polyubiquitin chain is regulated by the balance between the actions of ubiquitin ligases and deubiquitinating enzymes [45]; therefore, putative deubiquitinating enzymes possibly participate in the dynamic regulation of stalled translation surveillance. Taken together, it is tempting to hypothesize that K63 ubiquitination and deubiquitinating activities are regulated in response to the elongation rate of ribosomes even in regular translation, and an aberrant stall that cannot be resumed results in substantial elongation of a K63 polyubiquitin chain beyond a threshold to induce recruitment of factors for degradation and dissociation of stalled translation, as illustrated (S9 Fig).

In our study, the Hel2 depletion and K63R ubiquitin expression exhibited certain similarities in yeast. Both resulted in increased expression patterns of full-length proteins (Fig 3), and their temporal orders of action were closely related (Fig 6). Furthermore, both of these barely affected the quality control for nonstop translation (Fig 7 and S8 Fig). However, despite our efforts, the target substrates for the Hel2 E3 ubiquitin ligase and the K63 ubiquitin enzyme responsible for this stalled translation system remain to be identified, although our results strongly suggest that there is a direct connection between them.

In this report, among the factors tested, Asc1 was the only factor that was clearly indicated to function prior to Hel2 and K63 polyubiquitination. But, it is noteworthy that the luc2/Rluc ratio of the *not4∆hel2∆* double knockout strain was slightly, but significantly lower than that of the *hel2∆* strain (Fig 6B). Previously, Not4 was proposed to preserve translation elongation, as deletion of Not4 reduced the full-length protein from mRNA with stall signals [27]. In our study, the luc2/Rluc ratio of the asc1*∆* strain was lower than that of the hel2∆ strain and the K63R expressed strain (Fig 6B), suggesting the bidirectional role of Asc1 in protein synthesis; one is elimination of aberrant stalled translation by the quality control with Hel2 and K63 polyubiquitination, and the other is inhibition of translation through inefficient mRNA motifs such stall signals. Further, the fact that *not4∆asc1∆* strain could not be constructed suggests the overlapping roles of Asc1 and Not4 for the preservation of translation elongation. And thus it is worth mentioning that the Asc1/Not4 related pathway(s) could be distinct in some parts from the pathway Hel2 and K63 polyubiquitination are involved.

Previously, a possibility of a common mechanism for stalled translation during elongation (no-go type translation) and nonstop translation could not be ruled out because many of the factors, particularly a Dom34/Hbs1 complex, were involved in the quality controls of both aberrances [2]. In this study, our results suggested that the initial processes of the aberrant translation surveillances are distinct because K63R polyubiquitin affect no-go type stall translation more significantly than nonstop translation (Figs 2, 3, 7 and S8). Thus, we assume that aberrant translations are detected by a specific mechanism and are degraded by common mechanisms. In addition, we assume that K63 polyubiquitin is not significantly involved for the quality control of nonstop translation because nonstop is rather obvious for a surveillance system than a cumulative type of aberrance as no-go type stall translation. However, still more efforts are required to reveal the functional mechanism of K63 polyubiquitination in quality control pathways.

It is currently thought that ribosome dissociation, endonucleolytic cleavage, and/or the proteasomal degradation of a nascent protein should occur after an aberrant stall is detected [2]. Because our K63R mutant expressed full-length protein from mRNA with a stalled signal (Figs 2 and 3A) but did not affected the mRNA level (Fig 3B), we assume that K63 polyubiquitination is involved in inducing the ribosome dissociation or proteasomal degradation. Thus, identifying factors essential for regulating K63 polyubiquitination, such as substrates, receptors, and potential deubiquitinating enzymes of K63 polyubiquitination, will be critical for elucidating the detection mechanism of an aberrant stall and the regulatory relationships among the pathways that are involved at the site of stalled translation.

## Materials and Methods

### Strains and plasmids

The *S*. *cerevisiae* strains used in study are listed in S2 Table and the plasmids used are listed in S3 Table.

### Media

Yeast media were YPD or synthetic media, which were prepared with the appropriate dropout mix (ForMedium; Hunstanton, UK); 2% agar was added for plates. For analysis using MG132, yeast strains were grown in a synthetic medium, which included 0.17% yeast nitrogenous base without ammonium sulfate, 0.1% proline, 2% glucose, and 0.003% SDS with the appropriate dropout mix (ForMedium; Hunstanton, UK), as previously described [46]. Yeast cells were incubated for either 5 h for wild-type strain (BY4727) or 2 h for the *pdr5∆* strain (SKY142) after adding MG132 (Merck Millipore, Darmstadt, Germany); the concentrations used are indicated in the figures.

### Dual luciferase assay

Yeast cells were grown in appropriate media to log phase at 30°C and then collected by centrifugation. Whole cell lysates were prepared by vigorous shaking with glass beads on a FastPrep 24 (MP Biomedicals, Santa Ana, CA, USA). To determine luciferase activities, a Dual-Luciferase Reporter Assay System (Promega, Madison, WI, USA) and a GloMax 96 Microplate Luminometer (Promega, Madison, WI, USA) were used according to the manufacturer’s instructions.

### Western blotting

Whole cell lysates were prepared by vigorous shaking with glass beads on a FastPrep 24 (MP Biomedicals, Santa Ana, CA, USA). Lysates were separated by SDS-PAGE and then transferred to PVDF membranes (Merck Millipore, Darmstadt, Germany). Transferred proteins were probed using anti-Rluc (Merck Millipore, Darmstadt, Germany), anti-HIS3 (custom polyclonal antibody from Takara Bio, Shiga, Japan), or anti-PGK (Invitrogen, Carlsbad, CA, USA) antibodies, as indicated in the figures. Antibodies used for probing were detected with ImmunoStar LD (Wako, Osaka, Japan). Images were acquired with LAS-3000 (Fujifilm, Tokyo, Japan).

### Northern blotting

RNA was isolated by RNAiso Plus (Takara Bio, Shiga, Japan) with vigorous shaking with glass beads on a FastPrep 24 (MP Biomedicals, Santa Ana, CA, USA). Isolated RNA were separated by denaturing formaldehyde agarose gel and transferred to Hybond-N+ (GE Helthcare, Little Chalfont, UK). RNA was detected by DIG Northern Starter Kit (Roche Diagnostics, Basel, Switzerland). Images were acquired with LAS-3000 (Fujifilm, Tokyo, Japan). Customized oligo DNAs with 5' modification of digoxigenin were used as probes. Probes used in this report are: HIS3 probe [5'-CGACAACTGCGTACGGCCTGTTCGAAAGAT- 3'] and SCR1 probe [5'-ATCCCGGCCGCCTCCATCAC- 3'].

## Supporting Information

S1 Fig Genetic analysis using Hel2 and Ltn1 deletion strains. The plasmid harboring Rluc-HIS3 reporter, with either blank or CGAx12 signal, was introduced into the wild-type (WT), *hel2* ***∆***, and *ltn1* ***∆*** strains (BY4727, SKY61, S18-E01). Types of strains and reporters are indicated on the left. Transformant colonies were streaked on the SC- Tryptophan (Trp) (middle), and SC- Tryptophan (Trp), Histidine (His) (right) plates, and colony growth was monitored for 3 days at 30°C.
(TIF)

S2 Fig Dual luciferase assay for a Rluc-blank-luc2 reporter. Average luc2/Rluc ratios and standard deviations were determined from three independent measurements. (A) luc2/Rluc ratios of Rluc-blank-luc2 reporter from the wild-type (WT), *hel2* ***∆***, and *ltn1* ***∆*** strains (HRKW-1, SKY112, HRKW-5) and the wild-type stain incubated with MG132. Results for the wild-type strain (HRKW-1) were used as standard values, which were set to 100%. (B) Effects of ubiquitin arginine mutants on the Rluc-blank-luc2 reporter. The wild-type strain (HRKW-1) was used. Ubiquitins were expressed from plasmids. Ubi-WT indicates wild-type ubiquitin expressed from plasmid. (C) luc2/Rluc ratios of Rluc-blank-luc2 reporter from *asc1∆*, *dom34∆*, *hbs1∆*, *not4∆*, *ski3∆*, *rqc1∆ strain* (SKY114, HRKW-9, HRKW-3, SKY124, HRKW-7, SKY126).
(TIF)

S3 Fig Genetic colony growth test for the effects of ubiquitin mutants. Plasmids bearing wild-type and ubiquitin mutants were introduced into the strain harboring Rluc-HIS3 reporter (SKY26). Types of ubiquitin mutants are indicated on the left. Ubi-WT indicates wild-type ubiquitin. Transformant colonies were streaked on the SC- Uracil (Ura) (middle), and SC- Uracil, Histidine (Ura, His) (right) plates, and colony growth was monitored for 3 days at 30°C.
(TIF)

S4 Fig Effect of Hel2 deletion and K63R expression on different stall signals. Each stall signal was inserted at the junction of the Rluc and luc2 genes in the dual-luciferase reporter gene. PolyCGA indicates 12 repeats of CGA arginine codons, Polylysine indicates 12 repeats of AAG lysine codons, Polyarginine indicates 12 repeats of AGA arginine codons, PolyGGN indicates 12 repeats of GGN glycine codons (3 repeats of GGUGGCGGAGGG), and Polylysine (x24) indicates 24 repeats of AAG lysine codons. Reporter genes were introduced by plasmids into the wild-type (WT), *hel2∆*, or *ltn1∆* strains (BY4727, SKY61, S18-E01) or the wild-type strain expressing K63R ubiquitin from plasmid (K63R). Average luc2/Rluc ratios and standard deviations were determined from three independent measurements. *p < 0.05, **p < 0.01.
(TIF)

S5 Fig Anisomycin sensitivity based on expression of wild-type and mutant ubiquitins. (A) Wild-type and mutant ubiquitin plasmids were introduced into the wild-type strain (BY4727). Transformants were picked from SC-Ura plates and spotted on an SC-Ura + anisomycin (25 μg/ml) plate using 10-fold serial dilution. Ubi-WT indicates wild-type ubiquitin. Results were recorded after incubation for 3 days at 30°C. (B) The wild-type (WT), *hel2∆*, and *asc1∆* strains (BY4727, SKY61, S16-I04) were spotted on YPD and YPD + anisomycin (25 μg/ml) plates using 10-fold dilution. Results were recorded after incubation for 3 days at 30°C.
(TIF)

S6 Fig Azetidine-2-carboxylic acid (AZC) sensitivity of the *not4∆* strains. The wild-type (WT), *hel2∆*, *not4∆*, and *hel2∆not4∆* strains (BY4727, SKY61, SKY125, SKY151) were spotted on YPD and YPD + AZC (0.5 mg/ml) plates using 10-fold dilution. Results were recorded after incubation for 3 days at 30°C.
(TIF)

S7 Fig Temporal regulation of Asc1 with other factors for stalled translation. Average luc2/Rluc ratios and standard deviations were determined from three independent measurements. Asc1 was deleted in all strains. Thus, as examples, WT in this figure indicates the *asc1* ***∆*** single knockout strain, and *dom34* ***∆*** in this figure indicates the *asc1* ***∆*** *dom34* ***∆*** strain.
(TIF)

S8 Fig Effects of K63 polyubiquitination and Hel2 on the non-stop translation derived from hammerhead ribozyme. (A) A schematic image of the hammerhead ribozyme reporter with sTRSV hammerhead ribozyme. (B) Western blot for the Rluc protein expressed from the hammerhead ribozyme reporter gene. The reporter gene was introduced to yeast cells by plasmid. Empty vector (empty), wild-type ubiquitin (Ubi-WT), or K63R ubiquitin (K63R) plasmids were introduced into the wild-type, *ski3* ***∆*,** *hel2∆*, or *ltn1∆* strains (BY4727, S15-D07, SKY61, S18-E01). The expression of Rluc protein was detected using a Rluc antibody. PGK1 was used as a loading control. Ubi-WT indicates wild-type ubiquitin. Arrowhead 1 incidates the Rluc protein alone, and arrowhead 2 indicated full-length protein from the reporter gene.
(TIF)

S9 Fig Monitoring of stalled translation by the length of K63 polyubiquitin chain. (A) In normal translation elongation, the translational machinery is susceptible to both K63 polyubiquitination and deubiquitination, thus yielding a relatively short polyubiquitin chain at equilibrium. (B) In a situation of an aberrantly stalled translation, the deubiquilination step is hampered thus polyubiquitination overcomes, resulting in an extended K63 polyubiquitin chain. When K63 ubiquitin chain reaches a certain length, the chain is preferably detected by its receptor. Consequently, factors for dissociation and degradation are recruited and aberrant translation is eliminated.
(TIF)

S1 Table luc2/Rluc ratios with a CGAx12 reporter. (DOCX)

S2 Table luc2/Rluc ratios from reporter gens with different stall signals. (DOCX)

S3 Table Yeast strains used in this study. (DOCX)

S4 Table Plasmids used in this study. (DOCX)
