# Supplementary figures and images for: Inhibiting K63 Polyubiquitination Abolishes No-Go Type Stalled Translation Surveillance in Saccharomyces cerevisiae
Source: PLoS Genet. 2015 Apr 24;11(4):e1005197. doi: 10.1371/journal.pgen.1005197 (PMC4409330; doi:10.1371/journal.pgen.1005197)

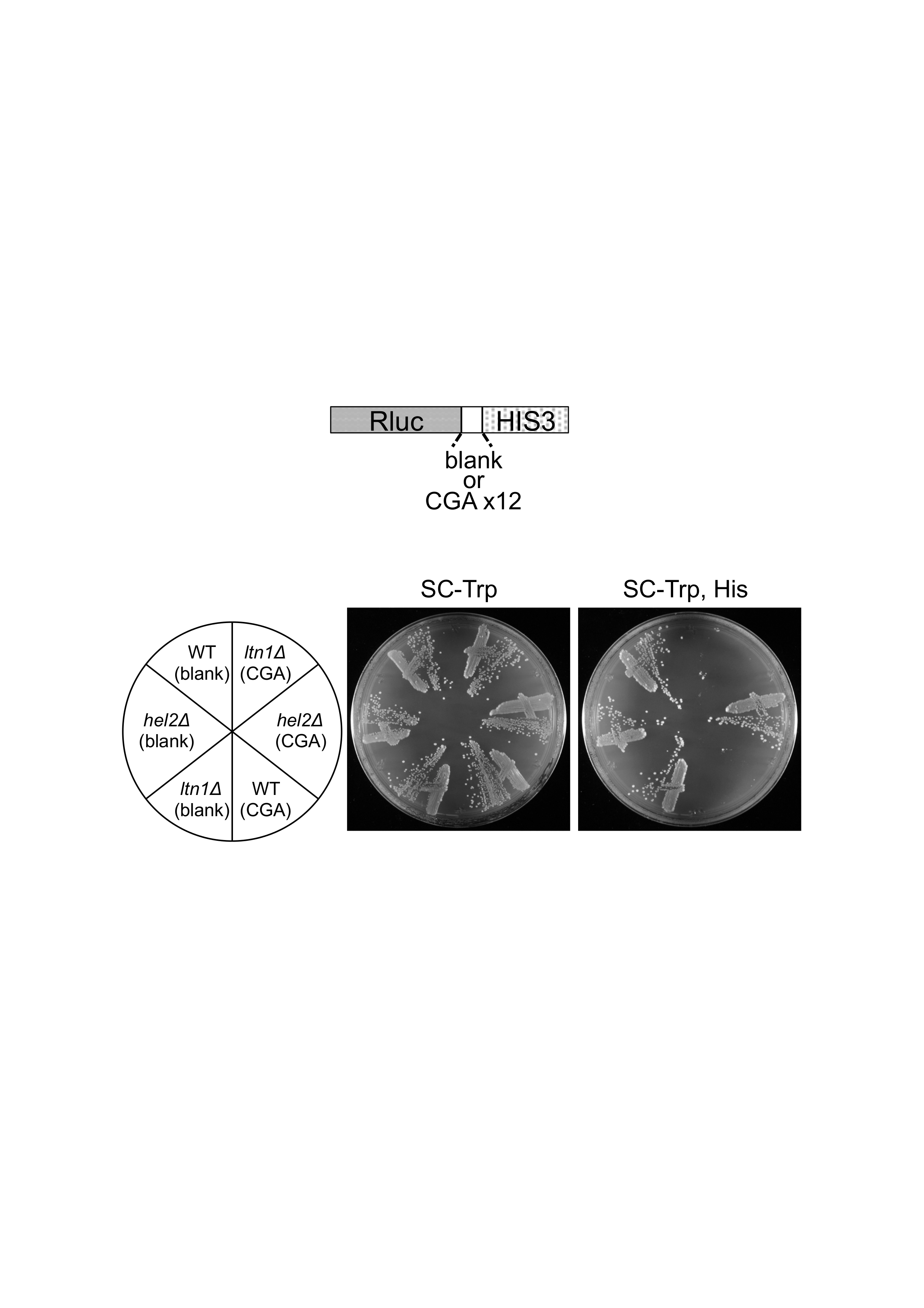

Supplement: S1 Fig — The plasmid harboring Rluc-HIS3 reporter, with either blank or CGAx12 signal, was introduced into the wild-type (WT), hel2 ∆, and ltn1 ∆ strains (BY4727, SKY61, S18-E01). Types of strains and reporters are indicated on the left. Transformant colonies were streaked on the SC- Tryptophan (Trp) (middle), and SC- Tryptophan (Trp), Histidine (His) (right) plates, and colony growth was monitored for 3 days at 30°C. (TIF) [file pgen.1005197.s001.tif]

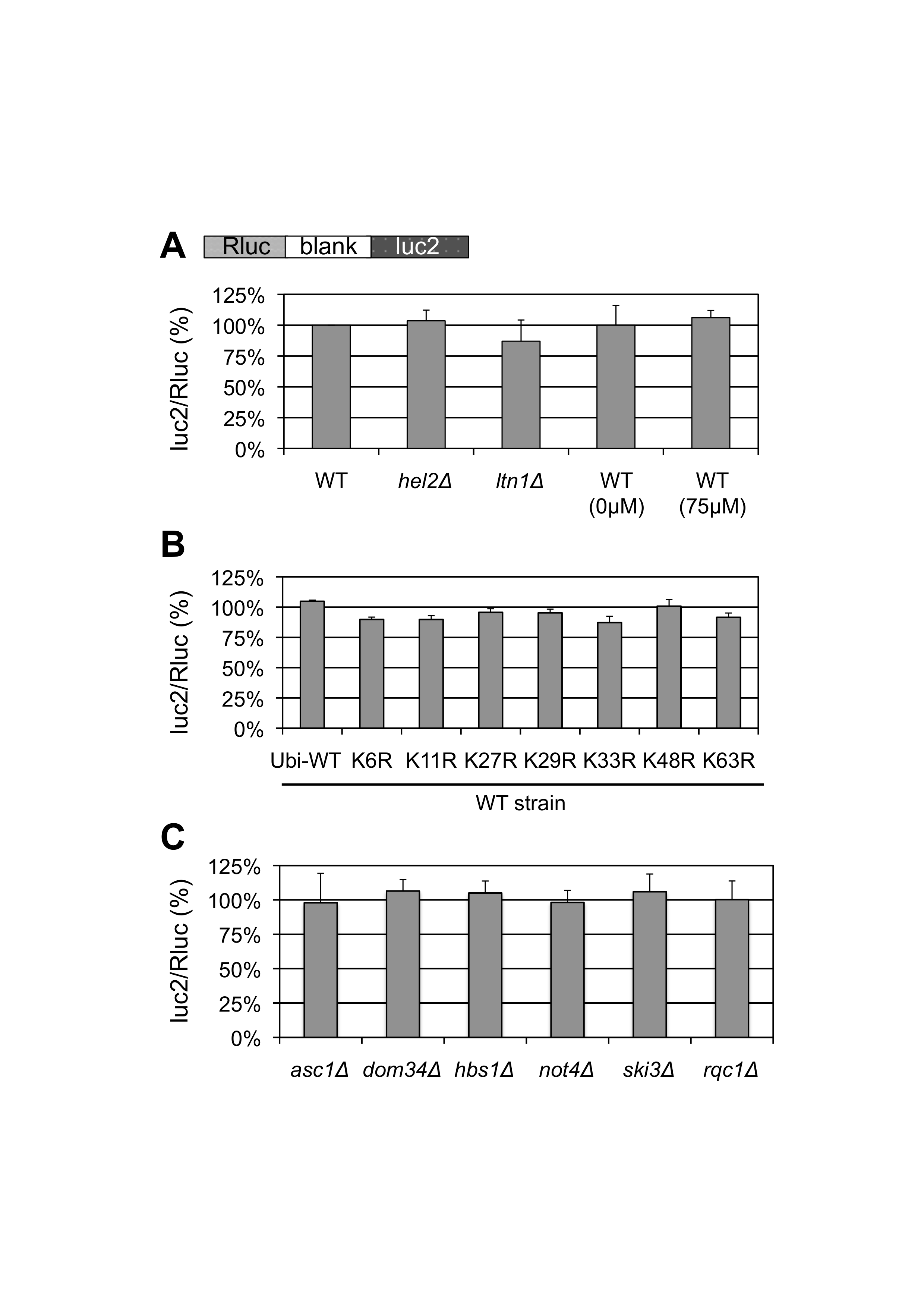

Supplement: S2 Fig — Average luc2/Rluc ratios and standard deviations were determined from three independent measurements. (A) luc2/Rluc ratios of Rluc-blank-luc2 reporter from the wild-type (WT), hel2 ∆, and ltn1 ∆ strains (HRKW-1, SKY112, HRKW-5) and the wild-type stain incubated with MG132. Results for the wild-type strain (HRKW-1) were used as standard values, which were set to 100%. (B) Effects of ubiquitin arginine mutants on the Rluc-blank-luc2 reporter. The wild-type strain (HRKW-1) was used. Ubiquitins were expressed from plasmids. Ubi-WT indicates wild-type ubiquitin expressed from plasmid. (C) luc2/Rluc ratios of Rluc-blank-luc2 reporter from asc1∆, dom34∆, hbs1∆, not4∆, ski3∆, rqc1∆ strain (SKY114, HRKW-9, HRKW-3, SKY124, HRKW-7, SKY126). (TIF) [file pgen.1005197.s002.tif]

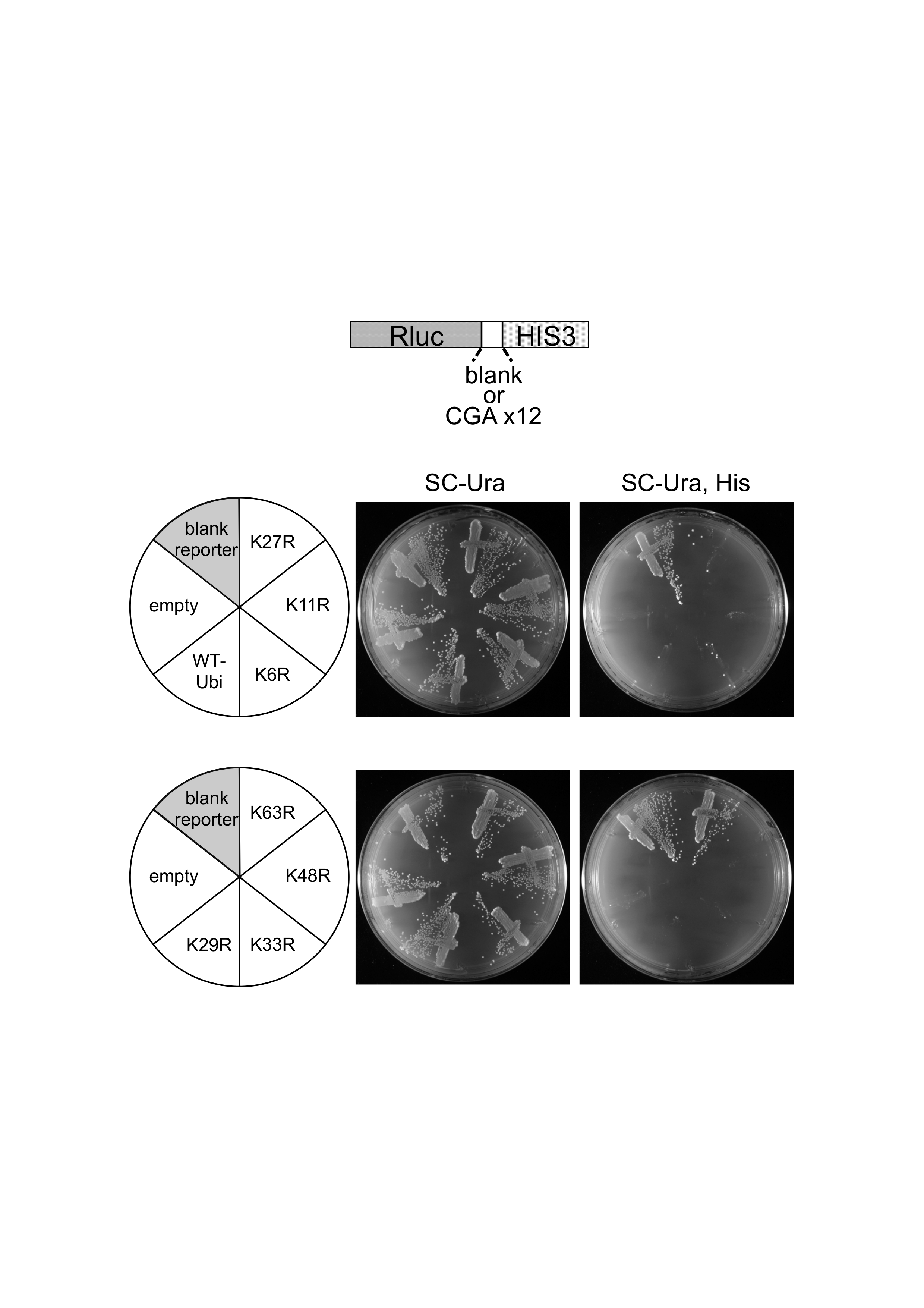

Supplement: S3 Fig — Plasmids bearing wild-type and ubiquitin mutants were introduced into the strain harboring Rluc-HIS3 reporter (SKY26). Types of ubiquitin mutants are indicated on the left. Ubi-WT indicates wild-type ubiquitin. Transformant colonies were streaked on the SC- Uracil (Ura) (middle), and SC- Uracil, Histidine (Ura, His) (right) plates, and colony growth was monitored for 3 days at 30°C. (TIF) [file pgen.1005197.s003.tif]

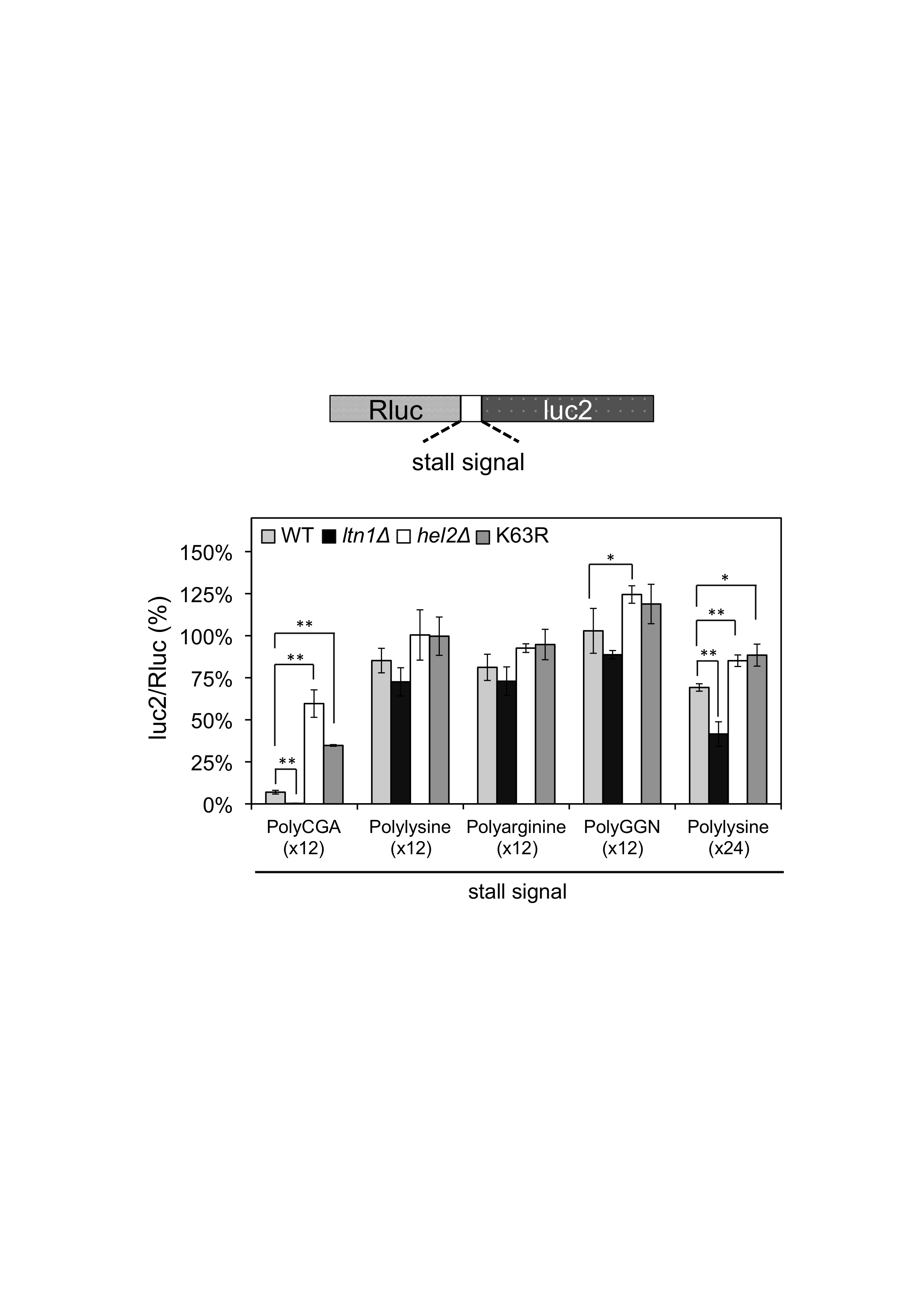

Supplement: S4 Fig — Each stall signal was inserted at the junction of the Rluc and luc2 genes in the dual-luciferase reporter gene. PolyCGA indicates 12 repeats of CGA arginine codons, Polylysine indicates 12 repeats of AAG lysine codons, Polyarginine indicates 12 repeats of AGA arginine codons, PolyGGN indicates 12 repeats of GGN glycine codons (3 repeats of GGUGGCGGAGGG), and Polylysine (x24) indicates 24 repeats of AAG lysine codons. Reporter genes were introduced by plasmids into the wild-type (WT), hel2∆, or ltn1∆ strains (BY4727, SKY61, S18-E01) or the wild-type strain expressing K63R ubiquitin from plasmid (K63R). Average luc2/Rluc ratios and standard deviations were determined from three independent measurements. *p < 0.05, **p < 0.01. (TIF) [file pgen.1005197.s004.tif]

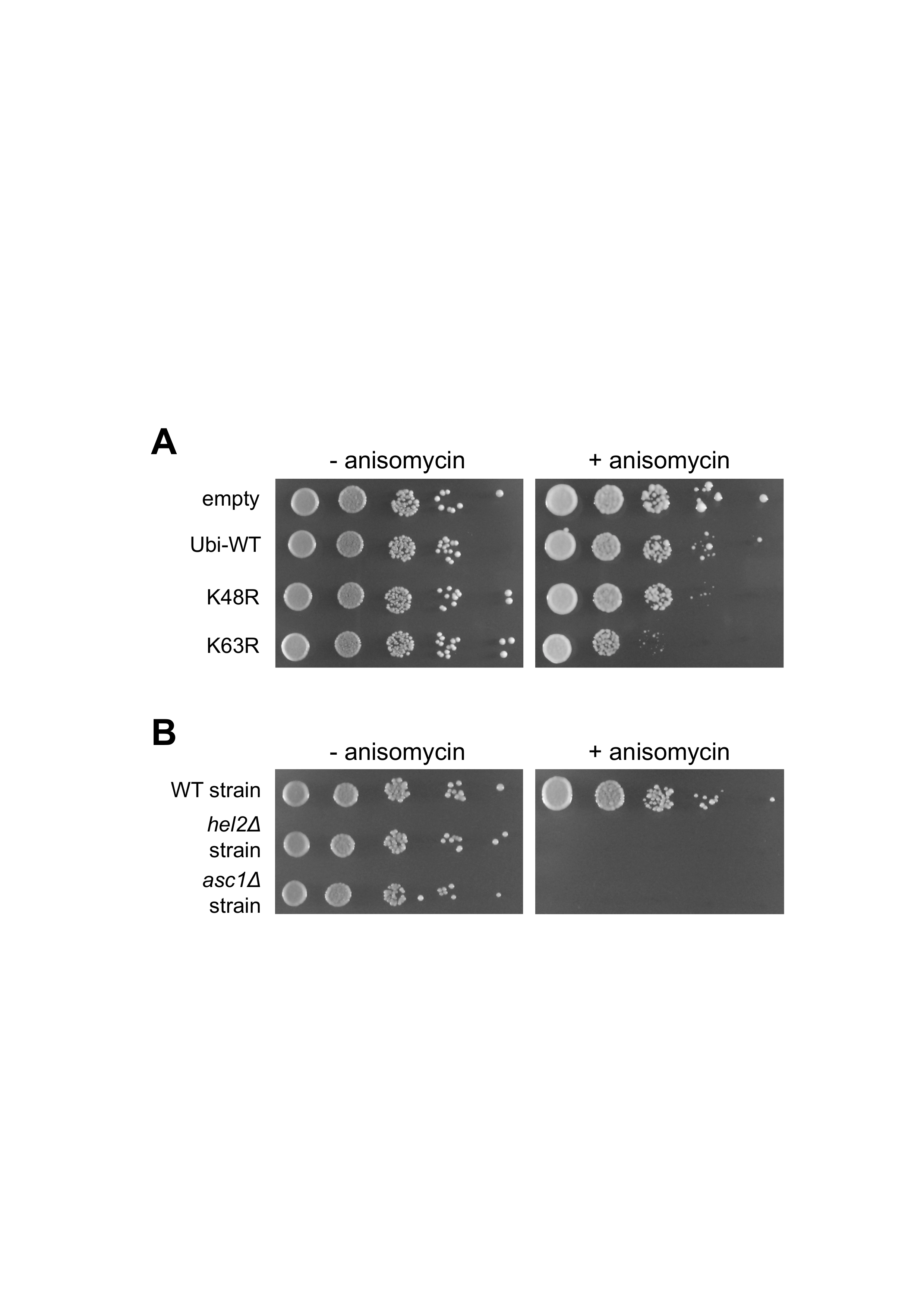

Supplement: S5 Fig — (A) Wild-type and mutant ubiquitin plasmids were introduced into the wild-type strain (BY4727). Transformants were picked from SC-Ura plates and spotted on an SC-Ura + anisomycin (25 μg/ml) plate using 10-fold serial dilution. Ubi-WT indicates wild-type ubiquitin. Results were recorded after incubation for 3 days at 30°C. (B) The wild-type (WT), hel2∆, and asc1∆ strains (BY4727, SKY61, S16-I04) were spotted on YPD and YPD + anisomycin (25 μg/ml) plates using 10-fold dilution. Results were recorded after incubation for 3 days at 30°C. (TIF) [file pgen.1005197.s005.tif]

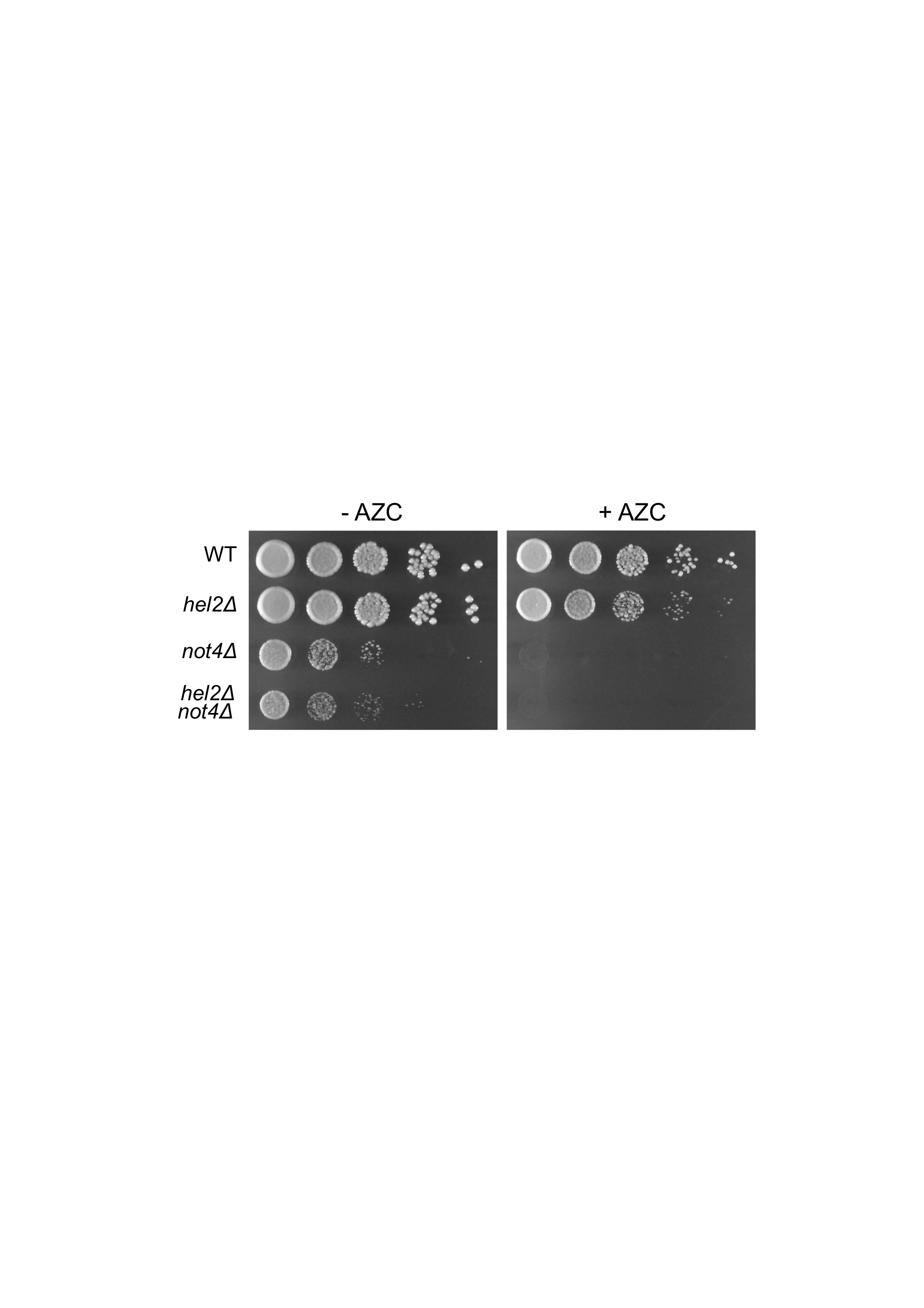

Supplement: S6 Fig — The wild-type (WT), hel2∆, not4∆, and hel2∆not4∆ strains (BY4727, SKY61, SKY125, SKY151) were spotted on YPD and YPD + AZC (0.5 mg/ml) plates using 10-fold dilution. Results were recorded after incubation for 3 days at 30°C. (TIF) [file pgen.1005197.s006.tif]

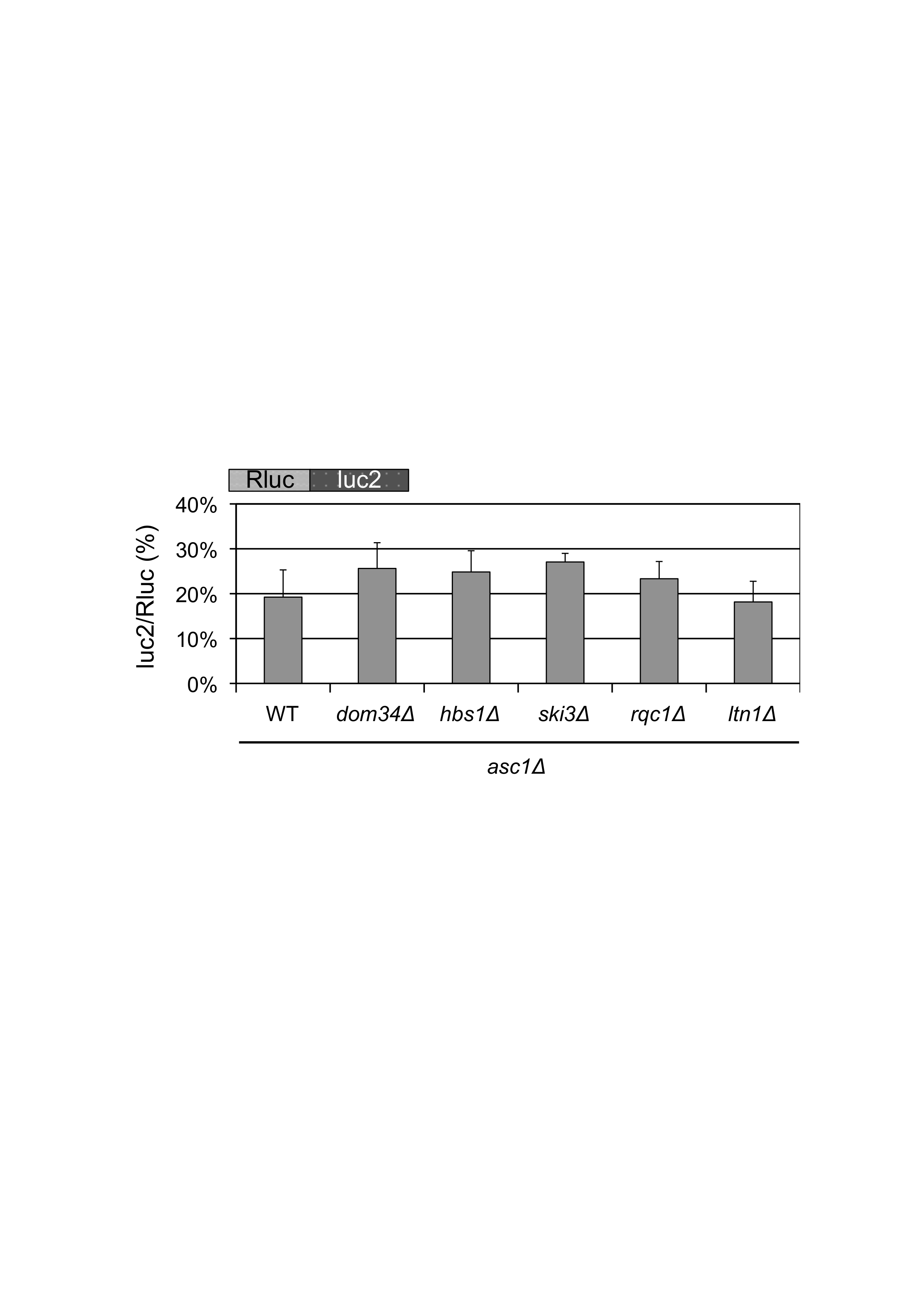

Supplement: S7 Fig — Average luc2/Rluc ratios and standard deviations were determined from three independent measurements. Asc1 was deleted in all strains. Thus, as examples, WT in this figure indicates the asc1 ∆ single knockout strain, and dom34 ∆ in this figure indicates the asc1 ∆ dom34 ∆ strain. (TIF) [file pgen.1005197.s007.tif]

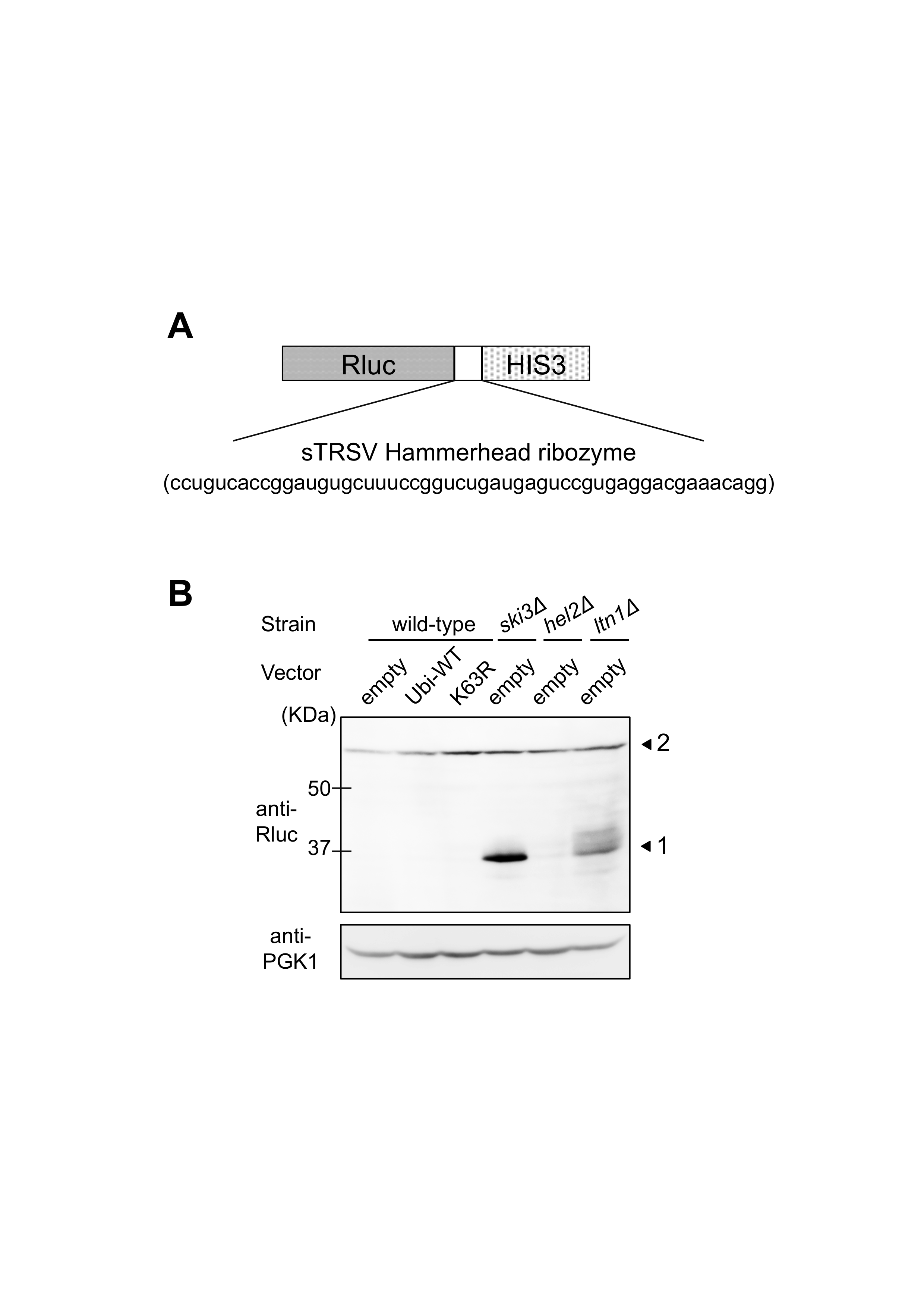

Supplement: S8 Fig — (A) A schematic image of the hammerhead ribozyme reporter with sTRSV hammerhead ribozyme. (B) Western blot for the Rluc protein expressed from the hammerhead ribozyme reporter gene. The reporter gene was introduced to yeast cells by plasmid. Empty vector (empty), wild-type ubiquitin (Ubi-WT), or K63R ubiquitin (K63R) plasmids were introduced into the wild-type, ski3 ∆, hel2∆, or ltn1∆ strains (BY4727, S15-D07, SKY61, S18-E01). The expression of Rluc protein was detected using a Rluc antibody. PGK1 was used as a loading control. Ubi-WT indicates wild-type ubiquitin. Arrowhead 1 incidates the Rluc protein alone, and arrowhead 2 indicated full-length protein from the reporter gene. (TIF) [file pgen.1005197.s008.tif]

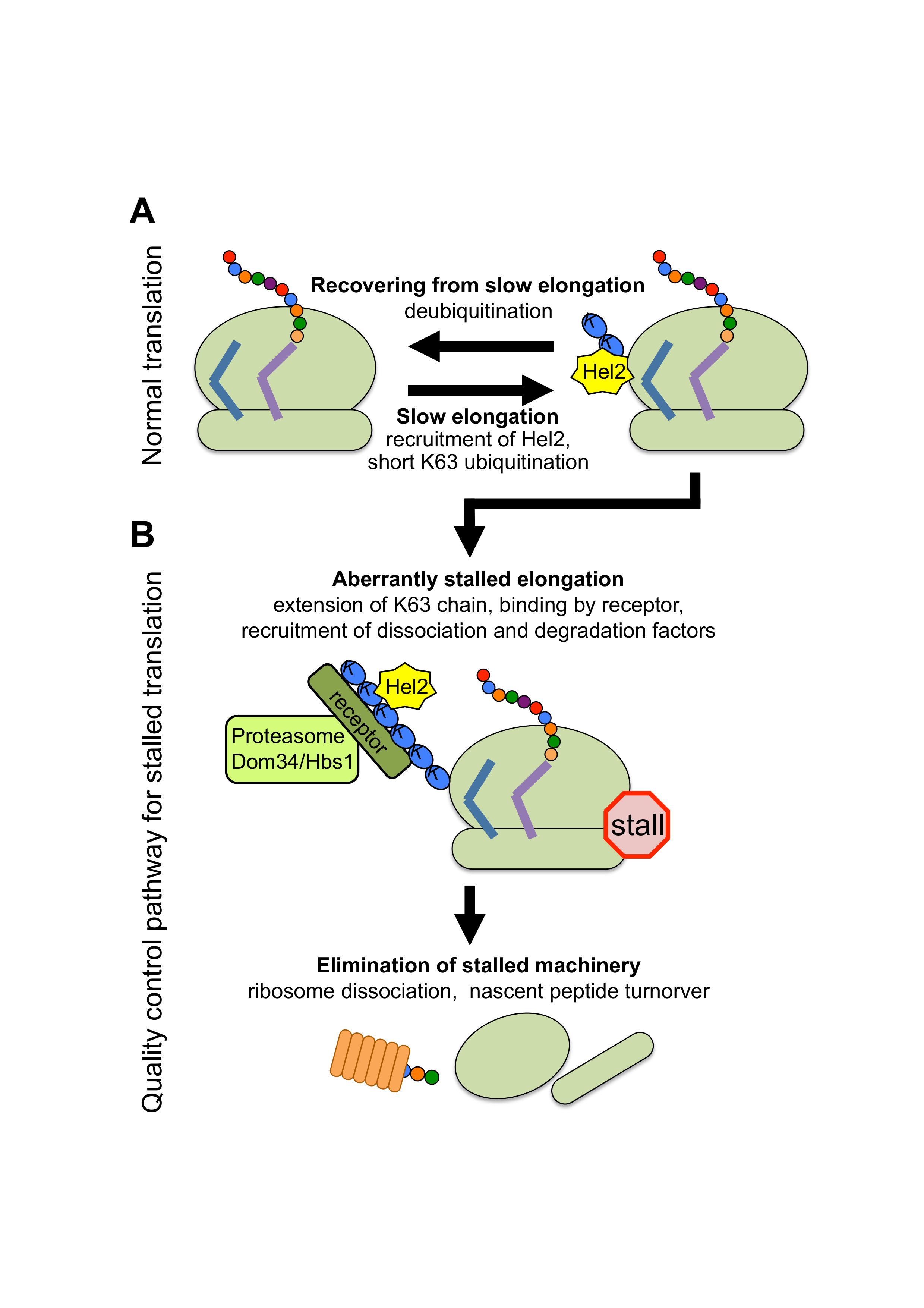

Supplement: S9 Fig — (A) In normal translation elongation, the translational machinery is susceptible to both K63 polyubiquitination and deubiquitination, thus yielding a relatively short polyubiquitin chain at equilibrium. (B) In a situation of an aberrantly stalled translation, the deubiquilination step is hampered thus polyubiquitination overcomes, resulting in an extended K63 polyubiquitin chain. When K63 ubiquitin chain reaches a certain length, the chain is preferably detected by its receptor. Consequently, factors for dissociation and degradation are recruited and aberrant translation is eliminated. (TIF) [file pgen.1005197.s009.tif]
