# Supplementary material for: Inhibiting K63 Polyubiquitination Abolishes No-Go Type Stalled Translation Surveillance in Saccharomyces cerevisiae
Source: PLoS Genet. 2015 Apr 24;11(4):e1005197. doi: 10.1371/journal.pgen.1005197 (PMC4409330; doi:10.1371/journal.pgen.1005197)
Supplement: S1 Table — (DOCX) [file pgen.1005197.s010.docx]

**S1 Table**

**luc2/Rluc ratios with a CGAx12 reporter**

| Figures | Strain | Additional conditions | luc2/Rluc (%) ± S.D.*^1,^ *^2^ | | |
| --- | --- | --- | --- | --- | --- |
| Fig. 1B | WT (HRKW-2) |  | 5.66 | ± | 0.84 |
| Fig. 1B | hel2∆ (SKY113) |  | 68.66 | ± | 6.22 |
| Fig. 1B | ltn1∆ (HRKW-6) |  | 0.24 | ± | 0.05 |
| Fig. 2C | WT (HRKW-2) | empty | 5.71 | ± | 1.22 |
| Fig. 2C | WT (HRKW-2) | UBI-WT | 8.09 | ± | 1.48 |
| Fig. 2C | WT (HRKW-2) | UBI-K6R | 9.15 | ± | 0.68 |
| Fig. 2C | WT (HRKW-2) | UBI-K11R | 8.92 | ± | 0.56 |
| Fig. 2C | WT (HRKW-2) | UBI-K27R | 8.19 | ± | 0.74 |
| Fig. 2C | WT (HRKW-2) | UBI-K29R | 6.54 | ± | 0.20 |
| Fig. 2C | WT (HRKW-2) | UBI-K33R | 6.73 | ± | 0.45 |
| Fig. 2C | WT (HRKW-2) | UBI-K48R | 8.76 | ± | 0.73 |
| Fig. 2C | WT (HRKW-2) | UBI-K63R | 39.70 | ± | 1.20 |
| Fig. 4A | WT (HRKW-2) | empty | 4.08 | ± | 0.93 |
| Fig. 4A | WT (HRKW-2) | UBI-K0 (singlecopy) | 2.99 | ± | 0.69 |
| Fig. 4A | WT (HRKW-2) | UBI-K0 (multicopy) | 13.23 | ± | 1.99 |
| Fig. 4A | WT (HRKW-2) | UBI-K63only (singlecopy) | 2.30 | ± | 0.59 |
| Fig. 4A | WT (HRKW-2) | UBI-K63only (multicopy) | 4.73 | ± | 1.31 |
| Fig. 5 | pdr5∆ (SKY142) | empty / DMSO (as 0μM) | 5.46 | ± | 0.99 |
| Fig. 5 | pdr5∆ (SKY142) | empty / MG132 (3μM) | 1.93 | ± | 0.80 |
| Fig. 5 | pdr5∆ (SKY142) | empty / MG132 (15μM) | 1.03 | ± | 0.52 |
| Fig. 5 | pdr5∆ (SKY142) | empty / MG132 (75μM) | 0.93 | ± | 0.24 |
| Fig. 5 | pdr5∆ (SKY142) | empty / PS341 (3μM) | 2.71 | ± | 0.49 |
| Fig. 5 | pdr5∆ (SKY142) | empty / PS341 (15μM) | 1.20 | ± | 0.38 |
| Fig. 5 | pdr5∆ (SKY142) | empty / PS341 (75μM) | 1.00 | ± | 0.34 |
| Fig. 5 | pdr5∆ (SKY142) | UBI-K63R / DMSO (as 0μM) | 31.80 | ± | 4.41 |
| Fig. 5 | pdr5∆ (SKY142) | UBI-K63R / MG132 (3μM) | 18.62 | ± | 2.01 |
| Fig. 5 | pdr5∆ (SKY142) | UBI-K63R / MG132 (15μM) | 8.91 | ± | 1.94 |
| Fig. 5 | pdr5∆ (SKY142) | UBI-K63R / MG132 (75μM) | 6.51 | ± | 1.28 |
| Fig. 5 | pdr5∆ (SKY142) | UBI-K63R / PS341 (3μM) | 25.09 | ± | 4.43 |
| Fig. 5 | pdr5∆ (SKY142) | UBI-K63R / PS341 (15μM) | 9.79 | ± | 2.39 |
| Fig. 5 | pdr5∆ (SKY142) | UBI-K63R / PS341 (75μM) | 7.22 | ± | 0.77 |
| Fig. 6A | WT (HRKW-2) | empty | 5.06 | ± | 0.36 |
| Fig. 6A | hel2∆ (SKY113) | empty | 59.88 | ± | 0.72 |
| Fig. 6A | asc1∆ (SKY115) | empty | 20.78 | ± | 3.14 |
| Fig. 6A | dom34∆ (HRKW-10) | empty | 13.11 | ± | 2.10 |
| Fig. 6A | hbs1∆ (HRKW-4) | empty | 8.85 | ± | 1.56 |
| Fig. 6A | not4∆ (SKY125) | empty | 8.76 | ± | 0.59 |
| Fig. 6A | ski3∆ (HRKW-8) | empty | 1.94 | ± | 0.02 |
| Fig. 6A | rqc1∆ (SKY127) | empty | 1.09 | ± | 0.22 |
| Fig. 6A | ltn1∆ (HRKW-6) | empty | 0.24 | ± | 0.05 |
| Fig. 6A | WT (HRKW-2) | UBI-K63R | 36.20 | ± | 6.37 |
| Fig. 6A | hel2∆ (SKY113) | UBI-K63R | 60.96 | ± | 1.03 |
| Fig. 6A | asc1∆ (SKY115) | UBI-K63R | 19.14 | ± | 3.87 |
| Fig. 6A | dom34∆ (HRKW-10) | UBI-K63R | 51.12 | ± | 4.64 |
| Fig. 6A | hbs1∆ (HRKW-4) | UBI-K63R | 37.57 | ± | 2.67 |
| Fig. 6A | not4∆ (SKY125) | UBI-K63R | 21.14 | ± | 1.92 |
| Fig. 6A | ski3∆ (HRKW-8) | UBI-K63R | 17.19 | ± | 4.87 |
| Fig. 6A | rqc1∆ (SKY127) | UBI-K63R | 11.80 | ± | 0.41 |
| Fig. 6A | ltn1∆ (HRKW-6) | UBI-K63R | 2.92 | ± | 0.41 |
| Fig. 6B | hel2∆ (SKY61) |  | 68.66 | ± | 6.22 |
| Fig. 6B | hel2∆ asc1∆ (SKY91) |  | 21.75 | ± | 1.64 |
| Fig. 6B | hel2∆ dom34∆ (SKY77) |  | 73.99 | ± | 2.95 |
| Fig. 6B | hel2∆ hbs1∆ (SKY76) |  | 68.88 | ± | 0.28 |
| Fig. 6B | hel2∆not4∆ (SKY151) |  | 57.00 | ± | 1.50 |
| Fig. 6B | hel2∆ ski3∆ (SKY62) |  | 73.33 | ± | 6.67 |
| Fig. 6B | hel2∆ rqc1∆ (SKY90) |  | 65.77 | ± | 15.10 |
| Fig. 6B | hel2∆ ltn1∆ (SKY79) |  | 59.46 | ± | 4.83 |
| Fig. S6B | WT (HRKW-2) | DMSO (as MG132 0μM) | 5.61 | ± | 0.77 |
| Fig. S6B | WT (HRKW-2) | MG132 (75μM) | 1.21 | ± | 0.09 |
| Fig. S9 | asc1∆ (S16-I04) |  | 19.23 | ± | 6.07 |
| Fig. S9 | asc1∆ dom34∆ (SKY97) |  | 25.62 | ± | 5.73 |
| Fig. S9 | asc1∆ hbs1∆ (SKY96) |  | 24.84 | ± | 4.74 |
| Fig. S9 | asc1∆ ski3∆ (SKY94) |  | 27.06 | ± | 1.92 |
| Fig. S9 | asc1∆ rqc1∆ (SKY93) |  | 23.33 | ± | 3.84 |
| Fig. S9 | asc1∆ ltn1∆ (SKY92) |  | 18.16 | ± | 4.60 |

*1. Standardized by the luc2/Rluc ratio of the blank reporter in a WT strain (HRKW-1 or BY4727).

*2. luc2/Rluc ratios are the means of 3 independent measurements.
