# Supplementary material for: Inhibiting K63 Polyubiquitination Abolishes No-Go Type Stalled Translation Surveillance in Saccharomyces cerevisiae
Source: PLoS Genet. 2015 Apr 24;11(4):e1005197. doi: 10.1371/journal.pgen.1005197 (PMC4409330; doi:10.1371/journal.pgen.1005197)
Supplement: S2 Table — (DOCX) [file pgen.1005197.s011.docx]

**S2 Table**

**luc2/Rluc ratios from reporter gens with different stall signals**

| Strain | stall signals | luc2/Rluc (%) ± S.D.*^1,^ *^2^ | | |
| --- | --- | --- | --- | --- |
| WT (BY4727) | PolyCGA | 6.97 | ± | 1.10 |
| WT (BY4727) | Polylysine | 85.22 | ± | 7.29 |
| WT (BY4727) | Polyarginine | 81.19 | ± | 7.79 |
| WT (BY4727) | PolyGGN | 102.90 | ± | 13.34 |
| WT (BY4727) | Polylysine (x24) | 69.23 | ± | 2.21 |
| ltn1∆ (S18-E01) | PolyCGA | 0.27 | ± | 0.14 |
| ltn1∆ (S18-E01) | Polylysine | 72.57 | ± | 8.38 |
| ltn1∆ (S18-E01) | Polyarginine | 72.95 | ± | 8.51 |
| ltn1∆ (S18-E01) | PolyGGN | 88.70 | ± | 2.50 |
| ltn1∆ (S18-E01) | Polylysine (x24) | 41.56 | ± | 7.27 |
| hel2∆ (SKY61) | PolyCGA | 59.64 | ± | 8.17 |
| hel2∆ (SKY61) | Polylysine | 100.44 | ± | 14.95 |
| hel2∆ (SKY61) | Polyarginine | 92.62 | ± | 2.56 |
| hel2∆ (SKY61) | PolyGGN | 124.50 | ± | 5.19 |
| hel2∆ (SKY61) | Polylysine (x24) | 85.13 | ± | 3.42 |
| WT (BY4727)  expressing UBI-K63R | PolyCGA | 34.76 | ± | 0.48 |
| WT (BY4727)  expressing UBI-K63R | Polylysine | 99.72 | ± | 11.39 |
| WT (BY4727)  expressing UBI-K63R | Polyarginine | 94.76 | ± | 9.05 |
| WT (BY4727)  expressing UBI-K63R | PolyGGN | 118.85 | ± | 11.72 |
| WT (BY4727)  expressing UBI-K63R | Polylysine (x24) | 88.46 | ± | 6.54 |

*1. Standardized by the luc2/Rluc ratio of the blank reporter in a WT strain (BY4727).

*2. luc2/Rluc ratios are the means of 3 independent measurements.
