# Supplementary material for: Inhibiting K63 Polyubiquitination Abolishes No-Go Type Stalled Translation Surveillance in Saccharomyces cerevisiae
Source: PLoS Genet. 2015 Apr 24;11(4):e1005197. doi: 10.1371/journal.pgen.1005197 (PMC4409330; doi:10.1371/journal.pgen.1005197)
Supplement: S3 Table — (DOCX) [file pgen.1005197.s012.docx]

**S3 Table**

**Yeast strains used in this study**

| Name | Genotype | Used in | Reference |
| --- | --- | --- | --- |
| BY4727 | *MATalpha his3∆200 leu2∆0 lys2∆0 met15∆0 trp1∆63 ura3∆0* | Fig. 1A, 3AB, S1, S3AB, S5, S6AB, S7AB, S8, S10 | Laboratory stock |
| SKY61 | *MATalpha his3∆200 leu2∆0 lys2∆0 met15∆0 trp1∆63 ura3∆0*  *hel2∆::kanMX* | Fig. 1A, 3AB, 6B, S1, S5, S7B, S8, S10 | This study |
| S18-E01 | *MATalpha his3∆200 leu2∆0 lys2∆0 met15∆0 trp1∆63 ura3∆0*  *ltn1∆::LEU2* | Fig. 1A, S1, S5, S10 | This study |
| HRKW-2 | *MATalpha his3∆200 leu2∆0 lys2∆0 met15∆0 trp1∆63 ura3∆0*  *HO::TEFp-Rluc-CGAx12-luc2-CYCt- kanMX* | Fig. 1B, 2C, 4A, 6A | This study |
| SKY113 | *MATalpha his3∆200 leu2∆0 lys2∆0 met15∆0 trp1∆63 ura3∆0*  *HO::TEFp-Rluc-CGAx12-luc2-CYCt- kanMX hel2::hphMX* | Fig. 1B, 6A | This study |
| HRKW-6 | *MATalpha his3∆200 leu2∆0 lys2∆0 met15∆0 trp1∆63 ura3∆0*  *HO::TEFp-Rluc-CGAx12-luc2-CYCt- kanMX ltn1∆::LEU2* | Fig. 1B, 6A | This study |
| SKY23 | *MATalpha his3∆200 leu2∆0 lys2∆0 met15∆0 trp1∆63 ura3∆0*  *HO::TEFp-Rluc-blank-HIS3-CYCt- kanMX* | Fig. 4B | This study |
| SKY24 | *MATalpha his3∆200 leu2∆0 lys2∆0 met15∆0 trp1∆63 ura3∆0*  *HO::TEFp-Rluc-CGAx12-HIS3-CYCt- kanMX* | Fig. 4B | This study |
| SKY142 | *MATalpha his3∆200 leu2∆0 lys2∆0 met15∆0 trp1∆63 ura3∆0*  *HO::TEFp-Rluc-CGAx12-luc2-CYCt-hphMX pdr5∆::kanMX* | Fig. 5 | This study |
| HRKW-4 | *MATalpha his3∆200 leu2∆0 lys2∆0 met15∆0 trp1∆63 ura3∆0*  *HO::TEFp-Rluc-CGAx12-luc2-CYCt- kanMX hbs1∆::hphMX* | Fig. 6A | This study |
| HRKW-8 | *MATalpha his3∆200 leu2∆0 lys2∆0 met15∆0 trp1∆63 ura3∆0*  *HO::TEFp-Rluc-CGAx12-luc2-CYCt- kanMX ski3∆::hphMX* | Fig. 6A | This study |
| HRKW-10 | *MATalpha his3∆200 leu2∆0 lys2∆0 met15∆0 trp1∆63 ura3∆0*  *HO::TEFp-Rluc-CGAx12-luc2-CYCt- kanMX dom34∆::hphMX* | Fig. 6A | This study |
| SKY115 | *MATalpha his3∆200 leu2∆0 lys2∆0 met15∆0 trp1∆63 ura3∆0*  *HO::TEFp-Rluc-CGAx12-luc2-CYCt- kanMX asc1∆::LEU2* | Fig. 6A | This study |
| SKY125 | *MATalpha his3∆200 leu2∆0 lys2∆0 met15∆0 trp1∆63 ura3∆0*  *HO::TEFp-Rluc-CGAx12-luc2-CYCt-hphMX not4∆:: kanMX* | Fig. 6A, S8 | This study |
| SKY127 | *MATalpha his3∆200 leu2∆0 lys2∆0 met15∆0 trp1∆63 ura3∆0*  *HO::TEFp-Rluc-CGAx12-luc2-CYCt-hphMX rqc1∆:: kanMX* | Fig. 6A | This study |
| SKY62 | *MATalpha his3∆200 leu2∆0 lys2∆0 met15∆0 trp1∆63 ura3∆0*  *hel2∆::kanMX ski3∆::hphMX* | Fig. 6B | This study |
| SKY76 | *MATalpha his3∆200 leu2∆0 lys2∆0 met15∆0 trp1∆63 ura3∆0*  *hel2∆::kanMX hbs1∆::hphMX* | Fig. 6B | This study |
| SKY77 | *MATalpha his3∆200 leu2∆0 lys2∆0 met15∆0 trp1∆63 ura3∆0*  *hel2∆::kanMX dom34∆::hphMX* | Fig. 6B | This study |
| SKY79 | *MATalpha his3∆200 leu2∆0 lys2∆0 met15∆0 trp1∆63 ura3∆0*  *hel2∆::kanMX ltn1∆::LEU2* | Fig. 6B | This study |
| SKY90 | *MATalpha his3∆200 leu2∆0 lys2∆0 met15∆0 trp1∆63 ura3∆0*  *hel2∆::hphMX rqc1∆::kanMX* | Fig. 6B | This study |
| SKY91 | *MATalpha his3∆200 leu2∆0 lys2∆0 met15∆0 trp1∆63 ura3∆0*  *hel2∆::hphMX asc1∆::kanMX* | Fig. 6B | This study |
| SKY151 | *MATalpha his3∆200 leu2∆0 lys2∆0 met15∆0 trp1∆63 ura3∆0*  *hel2∆::hphMX not4∆::kanMX* | Fig. 6B, S8 | This study |
| S17-A08 | *MATalpha his3∆200 leu2∆0 lys2∆0 met15∆0 trp1∆63 ura3∆0*  *HO:: TEFp-HIS3ns-HIS3t-kanMX* | Fig. 7ABC | This study |
| S17-A09 | *MATalpha his3∆200 leu2∆0 lys2∆0 met15∆0 trp1∆63 ura3∆0*  *HO:: TEFp-HIS3ns-HIS3t-kanMX ski3∆::hphMX* | Fig. 7ABC | This study |
| SKY123 | *MATalpha his3∆200 leu2∆0 lys2∆0 met15∆0 trp1∆63 ura3∆0*  *HO:: TEFp-HIS3ns-HIS3t-kanMX hel2::hphMX* | Fig. 7ABC | This study |
| SKY137 | *MATalpha his3∆200 leu2∆0 lys2∆0 met15∆0 trp1∆63 ura3∆0*  *HO:: TEFp-HIS3ns-HIS3t-kanMX ltn1∆::LEU2* | Fig. 7ABC | This study |
| HRKW-1 | *MATalpha his3∆200 leu2∆0 lys2∆0 met15∆0 trp1∆63 ura3∆0*  *HO::TEFp-Rluc-blank-luc2-CYCt- kanMX* | Fig. S2AB | This study |
| SKY112 | *MATalpha his3∆200 leu2∆0 lys2∆0 met15∆0 trp1∆63 ura3∆0*  *HO::TEFp-Rluc-blank-luc2-CYCt- kanMX hel2∆::hphMX* | Fig. S2A | This study |
| HRKW-5 | *MATalpha his3∆200 leu2∆0 lys2∆0 met15∆0 trp1∆63 ura3∆0*  *HO::TEFp-Rluc-blank-luc2-CYCt- kanMX ltn1∆::LEU2* | Fig. S2A | This study |
| HRKW-3 | *MATalpha his3∆200 leu2∆0 lys2∆0 met15∆0 trp1∆63 ura3∆0*  *HO::TEFp-Rluc-blank-luc2-CYCt- kanMX hbs1∆::hphMX* | Fig. S2C | This study |
| HRKW-7 | *MATalpha his3∆200 leu2∆0 lys2∆0 met15∆0 trp1∆63 ura3∆0*  *HO::TEFp-Rluc-blank-luc2-CYCt- kanMX ski3∆::hphMX* | Fig. S2C | This study |
| HRKW-9 | *MATalpha his3∆200 leu2∆0 lys2∆0 met15∆0 trp1∆63 ura3∆0*  *HO::TEFp-Rluc-blank-luc2-CYCt- kanMX dom34∆::hphMX* | Fig. S2C | This study |
| SKY114 | *MATalpha his3∆200 leu2∆0 lys2∆0 met15∆0 trp1∆63 ura3∆0*  *HO::TEFp-Rluc-blank-luc2-CYCt- kanMX asc1∆::LEU2* | Fig. S2C | This study |
| SKY124 | *MATalpha his3∆200 leu2∆0 lys2∆0 met15∆0 trp1∆63 ura3∆0*  *HO::TEFp-Rluc-blank-luc2-CYCt- hphMX not4∆::kanMX* | Fig. S2C | This study |
| SKY126 | *MATalpha his3∆200 leu2∆0 lys2∆0 met15∆0 trp1∆63 ura3∆0*  *HO::TEFp-Rluc-blank-luc2-CYCt- hphMX rqc1∆::kanMX* | Fig. S2C | This study |
| SKY25 | *MATalpha his3∆200 leu2∆0 lys2∆0 met15∆0 trp1∆63 ura3∆0*  *HO::GPDp-Rluc-blank-HIS3-CYCt- kanMX* | Fig. S4 | This study |
| SKY26 | *MATalpha his3∆200 leu2∆0 lys2∆0 met15∆0 trp1∆63 ura3∆0*  *HO::GPDp-Rluc-CGAx12-HIS3-CYCt- kanMX* | Fig. S4 | This study |
| SKY17 | *MATalpha his3∆200 leu2∆0 lys2∆0 met15∆0 trp1∆63 ura3∆0*  *HO::ADHp-Rluc-blank-HIS3-CYCt- kanMX* | Fig. | This study |
| SKY18 | *MATalpha his3∆200 leu2∆0 lys2∆0 met15∆0 trp1∆63 ura3∆0*  *HO::ADHp-Rluc-CGAx12-HIS3-CYCt- kanMX* | Fig. | This study |
| S16-I04 | *MATalpha his3∆200 leu2∆0 lys2∆0 met15∆0 trp1∆63 ura3∆0*  *asc1∆::kanMX* | Fig. S7B, S9 | This study |
| SKY92 | *MATalpha his3∆200 leu2∆0 lys2∆0 met15∆0 trp1∆63 ura3∆0*  *asc1∆::kanMX ltn1∆::LEU2* | Fig. S9 | This study |
| SKY93 | *MATalpha his3∆200 leu2∆0 lys2∆0 met15∆0 trp1∆63 ura3∆0*  *asc1∆::LEU2 rqc1∆::kanMX* | Fig. S9 | This study |
| SKY94 | *MATalpha his3∆200 leu2∆0 lys2∆0 met15∆0 trp1∆63 ura3∆0*  *asc1∆::kanMX ski3∆::hphMX* | Fig. S9 | This study |
| SKY96 | *MATalpha his3∆200 leu2∆0 lys2∆0 met15∆0 trp1∆63 ura3∆0*  *asc1∆::kanMX hbs1∆::hphMX* | Fig. S9 | This study |
| SKY97 | *MATalpha his3∆200 leu2∆0 lys2∆0 met15∆0 trp1∆63 ura3∆0*  *asc1∆::kanMX dom34∆::hphMX* | Fig. S9 | This study |
| S15-D07 | *MATalpha his3∆200 leu2∆0 lys2∆0 met15∆0 trp1∆63 ura3∆0*  *ski3∆::hphMX* | Fig. S10 | This study |
