# Supplementary material for: Inhibiting K63 Polyubiquitination Abolishes No-Go Type Stalled Translation Surveillance in Saccharomyces cerevisiae
Source: PLoS Genet. 2015 Apr 24;11(4):e1005197. doi: 10.1371/journal.pgen.1005197 (PMC4409330; doi:10.1371/journal.pgen.1005197)
Supplement: S4 Table — (DOCX) [file pgen.1005197.s013.docx]

**S4 Table**

**Plasmids used in this study**

| plasmid | Notes | Reference |
| --- | --- | --- |
| p414ADH | CEN6/ARSH4 TRP1 ADHp-MCS-CYCt | [47] |
| p414GPD | CEN6/ARSH4 TRP1 GPDp-MCS-CYCt | [47] |
| p416TEF | CEN6/ARSH4 URA3 TEFp-MCS-CYCt | [47] |
| p416GPD | CEN6/ARSH4 URA3 GPDp-MCS-CYCt | [47] |
| p414ADH Rluc-blank-HIS3 | CEN6/ARSH4 TRP1 plasmid carrying ADHp-Rluc-blank-HIS3-CYCt | This study |
| p414ADH Rluc-CGAx12-HIS3 | CEN6/ARSH4 TRP1 plasmid carrying ADHp-Rluc-CGAx12-HIS3-CYCt | This study |
| p414GPD Rluc-blank-HIS3 | CEN6/ARSH4 TRP1 plasmid carrying GPDp-Rluc-blank-HIS3-CYCt | This study |
| p414GPD Rluc-CGAx12-HIS3 | CEN6/ARSH4 TRP1 plasmid carrying GPDp-Rluc-CGAx12-HIS3-CYCt | This study |
| p416GPD Rluc-sTRSV  hammerhead ribozyme-HIS3 | CEN6/ARSH4 URA3 plasmid carrying GPDp-Rluc-sTRSV hammerhead ribozyme-HIS3-CYCt | This study |
| p416TEF Rluc-blank-luc2 | CEN6/ARSH4 URA3 plasmid carrying TEFp-Rluc-blank-luc2-CYCt | This study |
| p416TEF Rluc-CGAx12-luc2 | CEN6/ARSH4 URA3 plasmid carrying TEFp-Rluc-CGAx12-luc2-CYCt | This study |
| p416TEF Rluc-polylysine-luc2 | CEN6/ARSH4 URA3 plasmid carrying TEFp-Rluc-AAGx12-luc2-CYCt | This study |
| p416TEF Rluc-polyarginine-luc2 | CEN6/ARSH4 URA3 plasmid carrying TEFp-Rluc-AGAx12-luc2-CYCt | This study |
| p416TEF Rluc-polyGGN-luc2 | CEN6/ARSH4 URA3 plasmid carrying TEFp-Rluc-GGTGGCGGAGGG x3-luc2-CYCt | This study |
| p416TEF  Rluc-polylysine (x24)-luc2 | CEN6/ARSH4 URA3 plasmid carrying TEFp-Rluc-AAGx24-luc2-CYCt | This study |
| p416GPD UBI WT | CEN6/ARSH4 URA3 plasmid carrying  GPDp-UBI (first coding region from UBI4) -CYCt | This study |
| p416GPD UBI K6R | CEN6/ARSH4 URA3 plasmid carrying GPDp-UBI K6R -CYCt | This study |
| p416GPD UBI K11R | CEN6/ARSH4 URA3 plasmid carrying GPDp-UBI K11R –CYCt | This study |
| p416GPD UBI K27R | CEN6/ARSH4 URA3 plasmid carrying GPDp-UBI K27R –CYCt | This study |
| p416GPD UBI K29R | CEN6/ARSH4 URA3 plasmid carrying GPDp-UBI K29R –CYCt | This study |
| p416GPD UBI K33R | CEN6/ARSH4 URA3 plasmid carrying GPDp-UBI K33R –CYCt | This study |
| p416GPD UBI K48R | CEN6/ARSH4 URA3 plasmid carrying GPDp-UBI K48R –CYCt | This study |
| p416GPD UBI K63R | CEN6/ARSH4 URA3 plasmid carrying GPDp-UBI K63R -CYCt | This study |
| p416GPD UBI K0 | CEN6/ARSH4 URA3 plasmid carrying GPDp-UBI K0-CYCt | This study |
| p415GPD UBI K0 | CEN6/ARSH4 LEU2 plasmid carrying GPDp-UBI K0-CYCt | This study |
| p425GPD UBI K0 | 2-micron LEU2 plasmid carrying GPDp-UBI K0-CYCt | This study |
| p416GPD UBI K63only | CEN6/ARSH4 URA3 plasmid carrying GPDp-UBI K63only-CYCt | This study |
| p415GPD UBI K63only | CEN6/ARSH4 LEU2 plasmid carrying GPDp-UBI K63only -CYCt | This study |
| p425GPD UBI K63only | 2-micron LEU2 plasmid carrying GPDp-UBI K63only -CYCt | This study |
| p415GPD His-tagged UBI WT | CEN6/ARSH4 LEU2 plasmid carrying GPDp-His-tag-UBI-CYCt | This study |
| p415GPD His-tagged UBI K6R | CEN6/ARSH4 LEU2 plasmid carrying GPDp- His-tag-UBI K6R -CYCt | This study |
| p415GPD His-tagged UBI K11R | CEN6/ARSH4 LEU2 plasmid carrying GPDp- His-tag-UBI K11R –CYCt | This study |
| p415GPD His-tagged UBI K27R | CEN6/ARSH4 LEU2 plasmid carrying GPDp- His-tag-UBI K27R –CYCt | This study |
| p415GPD His-tagged UBI K29R | CEN6/ARSH4 LEU2 plasmid carrying GPDp- His-tag-UBI K29R –CYCt | This study |
| p415GPD His-tagged UBI K33R | CEN6/ARSH4 LEU2 plasmid carrying GPDp- His-tag-UBI K33R –CYCt | This study |
| p415GPD His-tagged UBI K48R | CEN6/ARSH4 LEU2 plasmid carrying GPDp- His-tag-UBI K48R –CYCt | This study |
| p415GPD His-tagged UBI K63R | CEN6/ARSH4 LEU2 plasmid carrying GPDp- His-tag-UBI K63R -CYCt | This study |
| p415GPD His-tagged UBI K0 | CEN6/ARSH4 LEU2 plasmid carrying GPDp- His-tag-UBI K0–CYCt | This study |
| p415GPD  His-tagged UBI K63only | CEN6/ARSH4 LEU2 plasmid carrying GPDp- His-tag-UBI K63only -CYCt | This study |

1. Mumberg D, Müller R, Funk M (1995) Yeast vectors for the controlled expression of heterologous proteins in different genetic backgrounds. Gene 156: 119-122.
